# Influence of Environment and Mitochondrial Heritage on the Ecological Characteristics of Fish in a Hybrid Zone

**DOI:** 10.1371/journal.pone.0005962

**Published:** 2009-06-18

**Authors:** Nicolas Stolzenberg, Bénédicte Nguyen The, Marie Dominique Salducci, Laurent Cavalli

**Affiliations:** University of Provence, UMR IMEP 6116, Marseille, France; University of Exeter, United Kingdom

## Abstract

**Background:**

Ecological characteristics (growth, morphology, reproduction) arise from the interaction between environmental factors and genetics. Genetic analysis of individuals' life history traits might be used to improve our understanding of mechanisms that form and maintain a hybrid zone.

**Methodology/Principal Findings:**

A fish hybrid zone was used to characterize the process of natural selection. Data were collected during two reproductive periods (2001 and 2002) and 1117 individuals (nase, *Chondrostama nasus nasus*, sofie *C. toxostoma toxostoma* and hybrids) were sampled. Reproductive dates of the two parental species overlapped at sympatric sites. The nase had an earlier reproductive period than the sofie; males had longer reproductive periods for both species. Hybridisation between female nase and male sofie was the most likely. Hybrids had a reproductive period similar to the inherited parental mitochondrial type. Growth and reproductive information from different environments has been synthesised following a bayesian approach of the von Bertalanffy model. Hybrid life history traits appear to link with maternal heritage. Hybrid size from the age of two and size at first maturity appeared to be closer to the size of the maternal origin species (nase or sofie).

Median growth rates for hybrids were similar and intermediate between those of the parental species. We observed variable life history traits for hybrids and pure forms in the different parts of the hybrid zone. Geometrical analysis of the hybrid fish shape gave evidence of two main morphologies with a link to maternal heritage.

**Conclusions/Significance:**

Selective mating seemed to be the underlying process which, with mitochondrial heritage, could explain the evolution of the studied hybrid zone. More generally, we showed the importance of studies on hybrid zones and specifically the study of individuals' ecological characteristics, to improve our understanding of speciation.

## Introduction

The arrival of a species in a new territory may have many consequences: modification of the environment, disappearance of certain species, maintenance of invasive species or hybridisation phenomena. According to Albert et al [1], interbreeding between distinct species can result in a variety of evolutionary outcomes (reinforcement (*sensu*Dobzhansky [2]), genetic extinction [3], [4], speciation [5], enhanced genetic diversity [6] and novel genetic combinations [7]–[10]. Different authors [9], [11]–[15] have shown the influence of natural hybridisation in animal evolution.

Hybridisation mechanisms are well known in cyprinids [16]–[18]. Several authors [19]–[21] have analysed the fitness, growth or survival rate of hybrids, mainly in artificial conditions. They have therefore measured the genetic component of postzygotic isolation [22]. More recently, several authors [1], [23]–[25] have estimated hybrid fitness in natural conditions. The genetic component of post-zygotic isolation is easier to measure than the ecological component, because it does not require a special environmental context. However, measurements of the genetic component reveal little about the forces giving rise to this component (i.e. natural selection or genetic drift). A growing number of analyses, generally based on samples taken directly from hybrid zones, have shown that there is an extrinsic or ecological component to natural selection, revealing different adaptations [26]. The outcome of natural hybridisation depends on the non-exclusive effects of both pre- and post-mating reproductive barriers [27]. In controlling the numbers of hybrids produced, premating reproductive barriers may play an important role in determining the genotype composition and fate of the hybrid zone [27]. The key to understanding the spatial patterning of hybridisation and the relative fitness of hybrids could lie in the ecology and breeding behaviour [28]. Environmental factors might also influence the level of hybridisation by reducing the relative reproductive success for one gender in one of the parental forms [29].

Reduced hybrid fitness may be partly caused by the disruption of co-adapted gene complexes [1]. Different studies have highlighted the importance of interactions between the mitochondrial and nuclear compartments, particularly for fitness [30], [31]. Mitochondria produce most of the energy by a process called OXPHOS (oxydative phosphorylation system). These organelles are consequently crucial to the proper growth and functioning of the cell [32]. But cellular metabolic energy production is critically dependent on nuclear mitochondrial interaction [33]. Indeed, the mitochondrion has its own genome, but more than 98 % of the proteins in this organelle are encoded by the nuclear genome; the expression of the nuclear and mitochondrial genomes must be coordinated [34].

Costedoat et al [35] recently described a hybrid zone in the River Durance, a tributary of the Rhône, between two species of cyprinids: *Chondrostoma nasus nasus* (Linnaeus, 1758), the nase, and *Chondrostoma toxostoma toxostoma*, the sofie. These authors demonstrated a phenomenon of introgressive hybridisation and the existence of a large number of viable genetic combinations.

*Chondrostoma n. nasus* originates from Central Europe. It recently increased its distribution range in France via the Rhine, using navigation canals constructed in the east and centre of France from 1860 onwards. It took around 40 years for the species to colonise all accessible French rivers [36]. In France, the sofie, *Chondrostoma t. toxostoma*, currently populates the Rhône catchment basin, Mediterranean rivers, the Garonne catchment basin, Atlantic rivers and the upstream part of the Loire catchment basin [37].

Studies of the ecological characteristics of hybrids and their comparison with those of parent species appear to be essential to our understanding of the origin and evolution of hybrid zones. Growth, reproduction and morphology are the principal ecological characteristics of the hybrids facilitating their comparison with the parent species and assessment of their survival potential and level of adaptation to the environment. All of these ecological traits are dependent on genetic and environmental factors. For example, even if a given species presents strong variability in growth, this characteristic remains within a given range, reflecting the role of genetic factors in controlling growth [38]. Other than intrinsic factors, temperature is the main factor affecting growth [39]. Moreover, analysis of individual shape is useful because it reflects the expression of both genetic and environmental factors [40]. In fish, morphometry is one of the simplest ways of assessing the evolutionary adaptation of a species to its environment [41]. Analyses of the reproductive traits of the parent species and of the hybrids should make it possible both to define the intervals of time where reproductive periods overlap and to determine which mating would be more sensible, therefore enabling us to understand some factors which might influence the fate evolution of the hybrid zone.

Here we focus on natural selection processes in the hybrid zone of *Chondrostoma* species in the Durance. This zone provides us with an opportunity to study certain ecological traits of hybrids and compare them with those of the parent species. We studied:

1. Growth, to determine the potential of the various combinations of hybrids observed by Costedoat et al [42] and compare them in different environments, based on the application of the Bayesian approach to the field of ichthyology;
2. Certain reproductive traits, making it possible to define reproductive periods and comparisons between different groups;
3. Morphology, to obtain an insight into the effects of genetics and environmental factors on individual phenotypes;
4. The mitochondrial DNA (mtDNA) type of each individual, making it possible to assess the relation of maternally inherited material with the growth, reproduction and morphology of individuals.

The extent of this hybrid zone and the variability of environmental conditions also made it possible to test whether the differences in ecological characteristics of different types of hybrids with respect to the parent species were similar in different environments. The results obtained should improve our understanding of the mechanisms underlying the maintenance and survival of hybrids and of the evolution of this hybrid zone. This work is novel in that it should provide information about the influence of maternal inheritance on the variability of individual ecological traits in the natural environment. Our results are of interest in terms of species conservation where hybridisation occurs.

## Materials and Methods

The genetic and morphometric data used in this work were already analysed and published by Costedoat et al [42]. The growth data and statistical analyses (in particular Bayesian approach) are unique to this article and in view of the growth results we reanalysed the morphometric data.

### Description of species and sites

The nase, *Chondrostoma nasus* originates from central Europe. It colonised the Rhone and Durance via navigation canals in the nineteenth and twentieth centuries. This species has rapid growth (max length>500 mm) and a reproductive period occurring around March–April at an age of around 3 years [36]. The sofie, *Chondrostoma toxostoma* is a smaller species (max length<250 mm) endemic to the South of France that reproduces in May-June from 2 years of age [43].

As hybridisation is frequent among Chrondrostoma species, it was necessary to identify each individual genetically. Each fish was identified by Costedoat et al [42] using four DNA markers and one mtDNA marker (cytochrome b). The four nuclear introns were: ribosomal protein gene S7 intron 1; triose phosphate isomerase 1b, Tpi1b intron 4; α-tropomyosin, α-Trop intron 5; and recombination activating gene 1, Rag 1 intron 2 (for more details see [42]).These markers were used to define the type (nase, sofie or hybrid) of each individual, and to determine the mtDNA type of each individual. Based on these markers, the following groups were identified: the parent species, *C. nasus* (N) and *C. toxostoma* (S), hybrids with *C. nasus* type mtDNA (HyN) and hybrids with *C. toxostoma* type mtDNA (HyS).

The fish studied were captured in the Durance River. Several hydroelectric power stations make use of this river, which is consequently separated into different sections by a number of dams. Fish were captured at different sites separated upstream and downstream by dams. Each site corresponded to several fishing sites separated by a maximum of 4 km. Four sites were defined. One was on one of the principal tributaries of the Durance, the Buech, and the other three were on the main course of the river at Manosque, Pertuis and Avignon (see Figure 9 [42]).

### Data collection

During years 2001 and 2002, 1117 fishes were collected by electric fishing (Héron, Dream Electronics). The fish were measured, photographed and a collection of oocytes or gonads, scales (for scalimetry) and a piece of tissue (genetic analysis) were sampled.

Fish were mostly caught between May and June, to ensure that the reproductive periods of each species overlapped. The age classes extended from one to six years, with a minimum of 35 and a maximum of 383 fish per age class. The fish could be assigned to four types at all four sites (Table 1).

**Table 1:** Number of fish caught.

	Buech	Manosque	Pertuis	Avignon
N	122	23		68
HyN	94	52	28	
HyS	31	72	10	
S	139	238	240	

Fish were caught during the reproductive period (March–June).

N = nase, S = sofie, HyN = hybrids with nase mtDNA, HyS = hybrids with sofie mtDNA.

### Morphology

**Figure 1:**
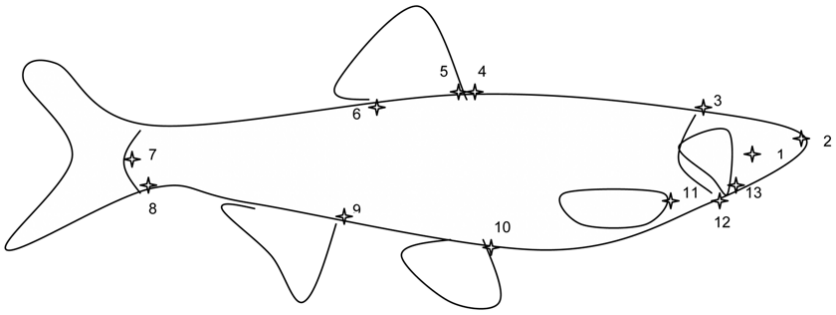
Positions of the landmarks used for morphetric analysis. Landmark description – 1: centre of the eye – 2: anterior extremity – 3: dorsal limit of head – 4: dorsal breakdown due to dorsal fin – 5: anterior dorsal fin insertion – 6: posterior insertion of dorsal fin insertion – 7: anterior extremity – 8: ventral insertion of caudal fin – 9: anterior anal fin insertion – 10: anterior ventral fin insertion – 11: anterior pectoral fin insertion – 12: ventral limit of head – 13: mouth insertion.

Fish were photographed with a Nikon Coolpix 995 camera and saved in the form of landmarks, using TPSdig software (Figure 1).

### Growth

Age of each fish was determined by scalimetry. The back-calculated fork length (in mm) was determined using the power model [44] for the 12 type-site groups of fish: nase-avignon (av-N), nase-buech (bu-N), nase-manosque (ma-N), sofie-buech (bu-S), sofie-manosque (ma-S), sofie-pertuis (pe-S), hybrids with nase mitochondrial origin-buech (bu-HyN), hybrids with nase mitochondrial origin-manosque (ma-HyN), hybrids with nase mitochondrial origin-pertuis (pe-HyN), hybrids with sofie mitochondrial origin-buech (bu-HyS), hybrids with sofie mitochondrial origin-manosque (ma-HyN), hybrids with sofie mitochondrial origin-pertuis (pe-HyN).

### Reproduction

In 2001, the sex of all individuals captured was determined and the gonadosomatic index (GSI) was calculated. The state of gonad maturity in females was assessed based on macroscopic and microscopic observations. In 2002, the same characters were assessed based on oocytes sampled through a cannula. The size at which maturity was attained in 50% of the population was determined based on a logistic regression model.

### Statistical analysis

#### Bayesian approach to the von Bertalanffy model

The von Bertalanffy model [45] is essentially an older version of the general model of growth proposed by Schnutte [46]. It is the preferred model when size is the variable studied and it is particularly appropriate and widely used in studies of fish [47]. It was used to model size, as a function of age for each of the 12 groups of fish defined on the basis of type-site combination:



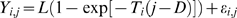



Where *Y_i,j_* and *ε_i,j_* define estimated size and its variability for fish *i* of age class *j*. The age classes correspond to whole years, from one to six. The parameters of the model are reproduction date (*D*, in years, beginning in January), adult size (*L*, in mm) of the group and the individual growth rate of fish *i* (*T_i_*). By contrast to the classical use of the von Bertalanffy model, we assumed here that the parameters were variable and consistent with the notion of a cohort and the variability observed between studies. In addition, to allow maximum flexibility we estimated the intrinsic growth rate individually as for a full model.

We used a hierarchical approach [48], because we distinguished three levels of variability:

1. When *L, T_i_,* and *D* are known, *Y_ij_* is normally distributed *L(1−exp[−T_i_ (j−D)])*, with a variance *σ^2^*.
2. When the mean and variance of each of the variable parameters (*L, T_i_, D*) are known, these parameters follow a normal distribution, N(*m_L_*, *σ^2^_L_*), N(*m_T_*, *σ^2^_T_*), N(*m_D_*, *σ^2^_D_*).
3. The parameters *m_T_* and *σ^2^_T_* correspond to the intrinsic growth rate of the group and its variability within the group. These two parameters are also assumed to be variable. When *μ_mT_* and *σ^2^_mT_* are known, *m_T_* is assumed to follow a log normal distribution (*μ_mT,_ σ^2^_mT_*). The *m_T_* parameter corresponds to the intrinsic growth rate of a type at a given site, and the use of a log normal distribution makes it possible to restrict its values to real, positive values [49].

Just as the mean terms are variable, so the variance terms, such as *σ^2^* and *σ^2^_T_* are assumed to be variable. They follow a non informative distribution defined according to Winbugs [50] (e.g. volume I: rats, a normal hierarchical volume) as the inverse of a gamma distribution (0.001, 0.001) for *σ^2^* and a squared standard distribution [0, 1] for *σ^2^_T_*.

Beyond the third level of variability, the fixed parameters are known as hyperparameters and their value is fixed by an expert or based on published results. The use of a Bayesian approach makes it possible to combine observational data with established known parameters for a species, to improve the estimation of growth curves. The values used for hyperparameters and the bibliographical references used are indicated in Table 2. For hybrids, there are no previous publications and the assumptions applying to the parent species were used to check for possible discrepancies in estimation. Similar results were obtained for the assumptions used. Growth curves were adjusted without taking sex into account because the data set for 2002 contained many unknowns. For the 2001 data, back-calculated sizes from age for each type showed that the data for male and female fish followed similar curves.

**Table 2:** Values of hyperparameters for prior distributions.

Parameters	Distribution	Hyperparameters
		**m_D_ N**: 3.5/12
D (year)	N(m_D_, σ^2^_D_)	**m_D_ S**: 545/12
		**σ_D_**: 1/12
		**m_L_ N**: 500
L (mm)	N(m_L_, σ^2^_L_)	**m_L_ S**: 200
		**σ_L_**:√(100 000)
m_T_	LN(μ_mT_, σ^2^_mT_)	**μ_mT_**: 0.26, **σ_mT_**: 0.5
*Ln(m_T_)*	*N(−2,0.6)*	

Nase (N) [59], [82], [83] and Sofie (S) [60], [61] values were defined according to previous publications.

The Bayesian approach estimates pre-distribution, based on the parameters of the model—the density of values taken by growth parameters for known sizes. This density cannot be calculated analytically, but can be simulated from prior distribution based on Monte Carlo-Markov chain algorithms [51]. The posterior distribution of each parameter was simulated with Winbugs 1.4 (cf. Appendix S1). For each group of 100,000 simulations with two initial chains (one based on prior mean values for the nase and the other based on prior mean values for the sofie), we were able to simulate pre-distributions after a burn-in phase of 10,000 simulations. Winbugs validation criteria were used to check that the chains mixed well. Autocorrelation problems were limited for pre-distribution parameters, by making use of only one of the four simulations carried out in the final analysis.

To fully understand the concept of *a posteriori* estimation we have used *T_i_* parameters. In the model definition the *T_i_* parameters were centred on the mean group value of growth rate with *a priori* value. Its standard deviation was derived from a non informative distribution which allowed large standard deviation values and thus, the normal distribution could be flat enough to allow a large range of potential values for simulation. Therefore, the estimation of *T_i_ a posteriori* (ie depending on the observed *Y_i_*) was no longer centred on the mean group but had moved toward a more probable value with respect to the observed *Y_i_*.

Median curves and 95% intervals for each group were calculated from the von Bertalanffy equation, using post-distribution quantile (respectively 2.5%, 50% and 97.5%) values for size at the different ages. For the group curves, *T_i_* was replaced by *m_T_* and for cohort curves (same group, same date of birth), the mean intrinsic growth rates from all individuals belonging to the cohort (∑_I = 1_^n^ T_i_/n) were used instead of *T_i_*. In order to state if the intrinsic growth rate of a cohort differed from the group, its difference with *m_T_* was estimated *a posteriori* using median values and its 95% intervals. Moreover, to visualise the impact of cohorts using conditional environment on fish growth curve, cohort growth curves were added to the graph of group curves. Only cohorts with enough sample size (cf. Appendix S2) and which differed visually from the group curve (estimated using all cohorts) were added.

#### Analysis of growth variances at one year

The first year of life may determine the subsequent development of the fish [52]. Size at one year reflects both the increase in size in the first year controlled by environment (site) and genetic type (type). We used the fork length, back-calculated for all fish, to ensure that the measure of size used was homogeneous. Age influences the back-calculated length, according to Lee's phenomenon [53], [54], in which the size of the oldest fish is underestimated. The final factor to consider is the notion of cohort. The fish captured belonged to seven different cohorts (1995 to 2001), according to their year of birth. The notion of a cohort only adds an effect of additional variability. This variability may be broken down into three terms: the effect of year of birth (intercept in equation 1) and, for a given birth year, the effect of site and type. A more complex model coupling site and type gave no significant improvement and was not retained. The analysis of variance model included two levels of variability: individual and cohort. It was a mixed effect model and could be modelled with the R package nlme [55]. It included fixed effects on mean back-calculated size (in bold) and variable effects on back-calculated size (in italics), see equation 1.

#### Equation 1

Size at one year of a fish (m) back-calculated from site (i), type (j) and age (k), taking cohort variability (l) into account. Fixed effects are shown in bold and variable effects are shown in italics. Individual variability is denoted ε.

Size_ijklm_ = **site-type_(ij)_**+**age_k_**+*cohort_l_*+*ε_ijklm_, with*

*cohort_l_ = intercept_l_+site_i/l_+type_j/l_*

*with the variable terms following normal distributions:*

*site_i/l_∼N(0, σ^2^_i_), type_j/l_∼N(0,σ^2^_j_), intercept_l_∼N(0,σ^2^) and ε_ijklm_∼N(0, σ^2^_individual_)*

#### Discriminant analysis of morphological data

Geometric analysis makes use of the notion of fish shape, in the form of a representation of points, invariant as a function of the position and size of the fish, and their measurement on photographs. Shapes are obtained using geometrical analysis and projection into the tangent space for the application of discriminant analysis to the 12 site-type groups [56], shapes package and the ADE4 protocol of R). Before carrying out discriminant analysis, we corrected for allometric effects within chondrostome types (nase, hybrids with nase mitochondrial origin HyN, hybrids with sofie mitochondrial origin HyS, sofie) by multivariate linear regression of the tangent co-ordinates of shapes as a function of fish size. As sofie and nase were known to have different adult sizes, we also adjusted the tangent coordinates from size (cf. Appendix S3). The tangent coordinates are synthetic variables and cannot be interpreted individually. [56] suggested studying changes in shape with deformation grids (package shapes of R). Two mean shapes were defined along each discriminant axes, associated respectively to negative and positive canonical scores, by using multivariate regression of the tangent coordinates upon the canonical score [57]. Based on the representation of this deformation grid between the two shapes, the distance between the points most sensitive to deformation were identified. Several key distances were found to distinguish between differences due to genetic type and/or environment (site). The discriminating power of the distances used was validated by Fisher's linear discriminant analysis [58].

## Results

### Reproductive date

#### Parent species overlap

Considering all sites together, the state of male and female maturity in the nase indicated a reproductive period potentially extending from the end of March to the end of April. For the sofie, the period extended from mid-April to the end of May, with the females tending to mature over a shorter period than the males (with an optimal reproductive period of about 10 days). Accordingly, the overlap favoured mating between nase female with sofie male.

#### Hybrid position

The smaller number of hybrids resulted in the collection of more sporadic information, particularly for males. The female HyN hybrids had a reproductive period similar to that of nase (end of March to end of April), whereas the female HyS hybrids had a reproductive period similar to that of the sofie (mid-April to end of May).

### Differences in size

#### Two distinct parental groups above two years old

**Figure 2:**
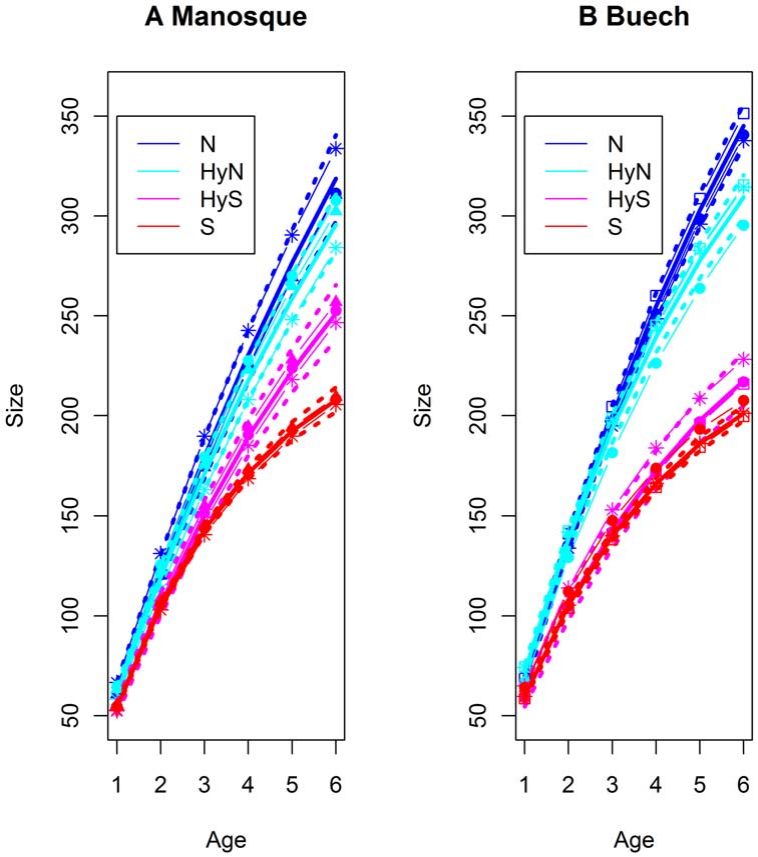
Median growth curves and 95% interval calculated from the von Bertalanffy model for all cohorts. Parameters of the model (adult size, intrinsic growth rate and date of reproduction) were estimated from the quantiles *of a posteriori* values generated by the Bayesian approach. N = nase, S = sofie, HyN = hybrids with nase mitochondria, HyS = hybrids with sofie mitochondria. Cohort growth curves of years 1997 to 2000 were added to the graphs: 2000 with full triangle, 1999 with full circle, 1998 with star and 1997 with empty square.

The median growth curves estimated with the von Bertalanffy model clearly distinguished the type at the age of two years old (Figure 2 for Buech and Manosque, Appendix S2 for all type, site and cohorts). At all sites, we observed two distinct parental growth curves: the lower one for sofie and the higher for nase. From age 2 and above, the 95% intervals of the two species growth curves were distinct and at age 3, the median size difference is around 40 mm.

#### Position of hybrid growth curves above two years old

**Figure 3:**
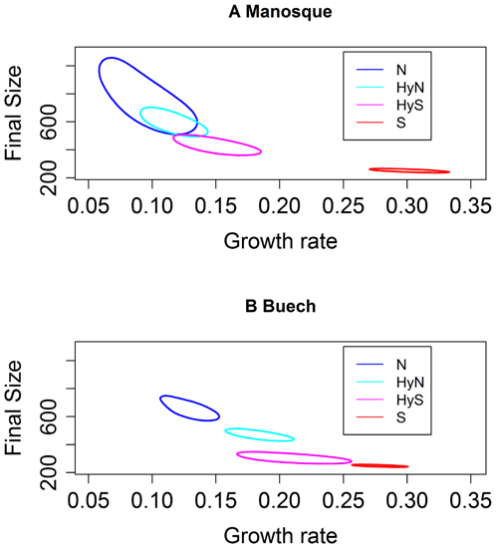
Isocontours of the growth parameters of the bayesian model. The isocontours for four types (nase, sofie, hybrids with nase mitochondria, hybrids with sofie mitochondria) were estimated for each site (Manosque and Buech) using a Gaussian node: the isocontour indicates the range of values around the median accounting for 95% of estimations.

The hybrid growth curves were between the two parental curves with a gradient from hybrids HyS to HyN. Hybrids HyN median size was higher than S and hybrids HyS as its 95% curve intervals were distinct above age 2, except in Pertuis. At the Pertuis site, the intrinsic HyN growth rate was twice lower than at the other site. Nevertheless, HyN hybrids exhibited growth curves and values near nase curves and above those of sofie and HyS hybrids (Figure 2 and 3). Similar observations could be applied to HyS hybrids which had growth curves and values near sofie and below those of hybrids HyN and nase.

#### Back-calculated size at one year

This method was used to refine the results obtained with the von Bertalanffy growth model. At one year old, the existence of a gradient in size of types (S, hybrids HyS, hybrids HyN,N) was not so clear and size differed between sites (Table 3). There was a significant interaction (log ratio between model with interaction and without: df = (28,22), L.ratio = 27.7, p-value = 1e-04) between types and sites (Avignon, Manosque, Pertuis, Buech). Depending on the site, multiple comparisons of size between types seemed to indicate two groups: N and HyN were never significantly different and neither were S and HyS. Hybrid mean sizes seemed closer to the inherited parental mitochondrial type (N or S). Moreover N and HyN showed similar conclusions for multiple t-test comparisons. They differed from HyT at Buech with a normal approximate p-value = 0.04 for nase and 0.007 for hybrids HyN, and from HyT and T at Manosque with a normal approximate p-value = 0.04 for nase and HyN hybrids and a normal approximate p-value = 0.03 for nase and similar for HyN hybrids.

**Table 3:** Back-calculated size at one year old.

	N	HyN	HyS	S	*σ*
***Avignon***	77±3.0				*0.08*
**Buech**	65±3.0	69±3.9	55±4.1	62±2.3	*2.3*
**Manosque**	63±5.1	59±3.1	51±2.1	50±2.4	*0.01*
**Pertuis**		49±7.0	64±6.0	58±3.4	*6.0*
*σ*	*2.7*	*2.0*	*0.03*	*0.01*	

Back-calculated size (in mm) adjusted for the analysis model used and expressed±the standard error. Standard errors were calculated as a function of the analysis model used, from the residuals, the variability within cohorts according to site (italic), type (italic) and the intercept (σ = 1×10^−2^). Interaction between sites and types were significant, results and multiple t-test comparisons are detailed in the text.

N = nase, S = sofie, HyN = hybrids with nase mtDNA, HyS = hybrids with sofie mtDNA.

#### Gradient in size at first reproduction from sofie to nase

Median size at reproduction was estimated by a logistic regression for all individuals at Buech (Table 4). We thus observed a gradient from the sofie, through HyS hybrids, followed by HyN hybrids, ending with the nase. Although incomplete, the data for the other sites were consistent with this gradient.

**Table 4:** Size at reproduction at the four sites studied.

	Buech				Manosque				Pertuis			Avignon
	N	S	HyN	HyS	N	S	HyN	HyS	S	HyN	HyS	N
Size (mm)												
M	210	119	151	125	-	107	-	108	106	186	-	234
F	232	122	198	121	-	96	-	80	108	198	-	-
F Fisher's Test												
≠ with	all	N	all	N		ns	ns		ns	ns		
		HyN		HyN								
M Fisher's test												
≠ with	all	N	N	N		ns	ns		HyN	S		

Sizes (median) were estimated from the logistic regression model. Fisher's test was applied separately to females (F Fisher's Test) and males (M Fisher's Test) to compare median sizes, all p-values were around 0.005 or non significant (ns) The table indicates where a type is significantly differente. N = nase, S = sofie, HyN = hybrids with nase mtDNA, HyS = hybrids with sofie mtDNA.

### Growth rate variability

#### Relative position of the four types

Regardless of the types of mitochondrial DNA present, hybrids had a similar median intrinsic growth rate (0.12 at Manosque, 0.19 at Buech) except for the Pertuis site (cf. Appendix S2 for all values). Hybrid median intrinsic growth rates were intermediate between those of the nase (0.09 at Manosque, 0.13 at Buech) and of the sofie (0.30 at Manosque, 0.28 at Buech). Whilst there was a clear gradient in terms of size, in terms of intrinsic growth rate the gradient existed for the parental species but was less pronounced for HyN hybrids to HyS hybrids (Figure 3). The 95% credible set overlapped between hybrids.

#### Type variability

Overall, variability in intrinsic growth rate was greater than variability in size, except for the nase at Manosque (Figure 3). The sofie displayed low levels of variability, whereas the nase and HyN hybrids were more variable for both the bayesian approach (Figure 3 and Appendix S2) and the linear growth model at one year (Table 3, ±3.7 and 2.6 cm, respectively for nase and hybrids HyN).

#### Site variability

The growth model used at one year old distinguished between the proportion of variability due to the species and that due to the environment (site) within cohorts (based on year of birth) (Table 3). The highest level of variability for growth during the first year was observed at the Pertuis site (±4.9), followed by Buech (±1.3). The bayesian approach also showed a high variabilty of HyS hybrids at Pertuis (Appendix S2).

#### Impact of cohorts

Based on the bayesian estimation, the intrinsic growth rates of some cohorts were distinct from the group at 95% but the differences were small and the impact on growth curves was limited (cf. Appendix S2 for all values). Overall, cohort growth curves could correspond to a group curve at 95% (Figure 2) and had no impact on the observed gradient in size or growth rate.

### Morphometry

**Figure 4:**
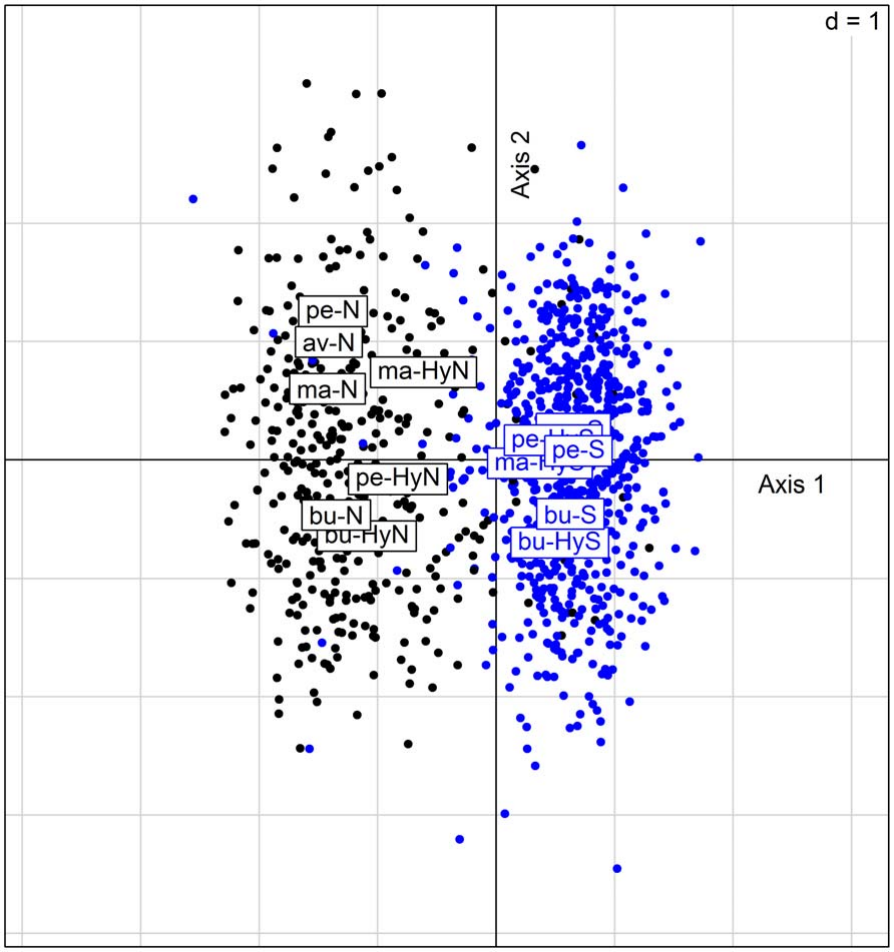
Discriminant analysis of fish shapes projected into the tangent space. Axes 1 and 2 account for 41.4 and 16%, respectively, of the variability between groups. Fish with sofie-type mitochondria are shown in blue, with nase-type in dark.

Discriminant analysis of fish shape as a function of group (av-N, bu-N, ma-N, bu-S, ma-S, pe-S, bu-HyN, ma-HyN, pe-HyN, bu-HyS, ma-HyN, pe-HyN) distinguished fish principally according to their mitochondrial origin (N or S; Figure 4). The first axis of the discriminant analysis accounted for 51.4% of variability between groups and separated fish according to mitochondrial origin; genetic information therefore appeared to be the most important. Information associated with site began to play a role from the second axis (accounting for only 16% of variability between groups) onwards. The correlation coefficient for the first axis showed that 43.4% of variation along this axis could be accounted for by the origin of the mitochondrial DNA, with the rest of the variation corresponding to residual intragroup variation.

**Figure 5:**
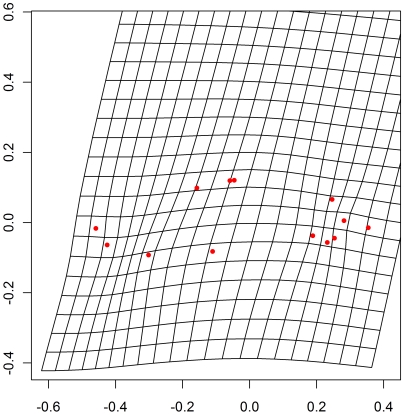
TPS grid deformation from mean shape along axis 1 of the discriminant analysis. i.e. from fish with sofie mitochondrial origin up to nase, the deformation is amplified by a magnitude coefficient of 4.

Transformations along axis 1 mostly concerned the ventral width of the fish and the length of the caudal fin as a proportion of total length (Figure 5). This transformation corresponded to the deformation of a quadrilateral based on points 9 (insertion of the anal fin), 11 (insertion of the pectoral fin), 4 (insertion of the dorsal fin) and 7 (end of the lateral line) (Figure 1). This quadrilateral is defined using the distances between points 9 and 7, 9 and 4 and 9 and 11, divided by total length (the distance between points 2 and 7). This analysis generates three coefficients of allometry, the mean values of which seemed to distinguish between the two classes of fish defined by their mitochondrial origin. Therefore a Fisher's discriminant analysis was carried out with these three distance variables and the two mitochondrial classes. Results are shown in Table 5; information for subgroups (hybrids and parental species for each mitochondrial class) was also given. Fisher's discriminant analysis classified the fish with percentages of correct classification greater than 90%: 93.4% and 93.8% of fish correctly classified by cross-validation and by classification of fish in 2002 based on 2001 data, respectively. Details of this analysis as a function of mitochondrial origin and also for subgroups are provided in Table 5. Correct classification percentages for parental types exceeded 95%, whereas in 2002 those for HyN hybrids and HyS hybrids were only about 77%.

**Table 5:** Discriminant analysis for determining mitochondria origin from morphology.

Mitochondria	Mean (σ) allometric coefficient			% correctly classified	
	9_7	9_4	9_11	CV	2002
**Nase:**	0.27 (0.016)	0.28 (0.022)	0.46 (0.03)	87.2	90.0
*N*	*0.27*	*0.29*	*0.47*	*96.7*	*97.1*
*HyN*	*0.27*	*0.27*	*0.45*	*77.6*	*78.0*
**Sofie:**	0.29 (0.016)	0.25 (0.013)	0.43 (0.019)	93.4	95.9
*S*	*0.29*	*0.25*	*0.43*	*97.9*	*97.9*
*HyS*	*0.28*	*0.25*	*0.43*	*91.8*	*75.7*

The mean and standard deviations of allometric coefficients used and the quality of classification by the linear model, as assessed by cross-validation (CV) or by calculating data for 2002 based on data for 2001.

N = nase, S = sofie, HyN = hybrids with nase mtDNA, HyS = hybrids with sofie mtDNA.

## Discussion

### Characteristics of the parent species

In the Durance River, the nase displays higher growth rates than the sofie, as in allopatric conditions [59], [60]. The maximum size observed in our study was 49 cm for the nase (50 cm in the study by Nelva [36]) and 22 cm for the sofie (24 cm in the study by Gozlan [61]). Maturity was acquired at the age of about three years in the nase and about two years for the sofie, one year earlier than [36] for the nase and [61] for the sofie.

In the Durance, the female nase had an optimal reproductive period of about three weeks at the end of March and beginning of April. Keith and Allardi [37] reported reproduction during a three-week period in March and April (water temperatures of 8 to 11°C) at shallow sites with a strong current, with the eggs laid on gravel or large pebbles.

Female sofie in the Durance had an optimal reproductive period of about 10 days during the month of May. According to Gozlan [61], female sofie laid their eggs at the end of May or start of June, some 30 to 40 days after the nase, on gravel in small tributaries or on stones in principal water courses.

In contrast to published observations, particularly [61], we observed an overlap in the Durance between the zones and periods of reproduction of the two species. In addition, as in many other species, males began to mature earlier and over a longer period than females. These phenomena make hybridisation possible. Results for reproductive periods of the parent species reveal that certain combinations were more likely than others. Hybridisation between female nase and male sofie was the most likely because the female nase matured earlier than the female sofie and the male sofie had a longer, earlier period of maturity than the female sofie.

### Ecological characteristics of hybrids and evolution of the hybrid zone

The partial overlap in reproductive periods and zones of nase and sofie in the Durance has generated hybridisation. However, hybridisation can only be maintained if the hybrids, or certain hybrid combinations, are viable and if their ecological characteristics do not result in their being less fit than the parent species. The ecological characteristics of hybrids may be influenced by intrinsic factors (complex interactions between parental genomes [6]) or by environmental factors.

One frequently cited reason for the poor adaptation of hybrids is difficulty feeding. Such problems occur when the parental types have different alimentary specialisations of morphological types, as is the case for benthic and limnetic sticklebacks [62] or whitefishes [63]. By contrast to these examples of alimentary counterselection, the isotopic values obtained for the nase, sofie and hybrids did not differ significantly (Durbec et al 2007, Symposium on Non-Native Fishes of the Fisheries Society of the British Isles).There is therefore no evidence for different alimentary specialisations between these types.

Hybrid viability observed in the sampled fish was demonstrated by the presence of five- to six-year-old hybrids. Most of the time, hybrid growth was intermediate between the growth of the two parental species. Examples of natural hybrids with a higher relative fitness exist (review in [1]), but no heterosis effect was observed on hybrids in this study. Growth, considered as an integral factor, demonstrated that the habitat was adequate and highlighted the high level of adaptation of the hybrids caught. These results differed from those of Hatfield [64], who found that F1 hybrid sticklebacks grew less rapidly than the parent species, and those of Hagen and Taylor [65], who demonstrated that intermediate hybrid phenotypes were disadvantaged in the parental niche.

The diversity of life history traits in hybrids is often accounted for by the diversity of possible associations of different parts of the parent genome by recombination [6], [13]. For wild caught specimens, different combinations of nuclear parental genetic information would coexist and could influence life history traits. But, an analysis of the ecological characteristics of the different groups showed that for the ecological characteristics studied (growth, reproduction and morphology), hybrids showed more similarities to the inherited parental mitochondrial type. If we consider the different genetic combinations, the von Bertalanffy growth curves allowed a gradient in size after two years of age: nase, HyN hybrids, HyS hybrids and sofie. Hybrid growth most closely matched that of the parent with the same mitochondrial origin. Size at first reproduction and reproductive periods of the hybrids were also closer to those of the inherited parental mitochondrial type. Eventually, discriminant analysis on shape tangent coordinates indicated two distinct shapes depending on maternal mitochondrial inheritance (Figure 4).

Therefore, ecological characteristics seemed related to maternal inheritance. Even if the involvement of nuclear gene could not be ruled out, it appeared more likely to be a mitochondrial influence on hybrid recombination and/or on hybrid phenotypes.

The role of maternally inherited mitochondrial genomes in hybrids has not been widely examined [66]. Indeed, mitochondrial DNA contains several important genes able to interact with the nuclear genome. Notably the OXPHOS system complexes (five-multi subunit enzymes complexes) consist of both mitochondrial and nuclear polypeptides [67]. For example a single amino acid substitution in cytochrome c from copepods *Trigriopus californicus* has had a detrimental effect on complex IV activity of OXPHOS (key aspect of mitochondrial bioenergetics) [68]. Other authors have also demonstrated that fitness loss in copepod hybrids by reduced ATP synthesis by OXPHOS was linked to nuclear-mitochondrial interactions [66]; maternal backcross hybrids have shown a recovery of the OXPHOS system and concomitant recovery of fitness, suggesting that mitochondrial function may be correlated with fitness. Another study [69] was consistent with a causative role for mtDNA variations in phenotypic differences (egg size, fecundity, cold resistance and starvation resistance), among fly line. However, the authors did not completely rule out the involvement of nuclear genes.

Based on the work of Costedoat et al [42], HyN hybrid combinations with a larger number of nuclear N markers are more frequent than expected under Hardy-Weinberg equilibrium (Table 6). The same applied although less pronounced for HyS hybrid combinations. Indeed, according to observed maturity state, HyN hybrids had reproduction dates most similar to those of the nase, favouring the introgression of nase genes. In the same way, morphometric analysis showed that the nase and HyN hybrids were closer to the “cruiser” type described by Webb and Weihs [70], enabling them to swim and to maintain their position in upper currents [71]. This ability may be important during reproduction, because the nase reproduces in more rapid currents than the sofie [72].

**Table 6:** Genetic combinations of fish with respect to Hardy-Weinberg equilibrium.

			mtDNA marker			
Number of nDNA markers:			N		S	
Same as mtDNA	Hybrids	Different from mtDNA	2001	2002	2001	2002
**4**			**+**	**+**	**+**	**+**
**3**	1		**+**	**+**		**−**
**3**		1	**+**	**+**	**+**	
**2**	2				**−**	**−**
**2**		2	**+**			
**2**	1	1			**−**	**−**
**1**	3					
**1**		3				
**1**	2	1				
**1**	1	2				
	3	1				
	1	3	**−**	**−**		
	2	2	**−**			
	4				**−**	**−**
		4			**+**	**+**

Genetic combinations of over-represented (+) or under-represented (−) fish with respect to Hardy-Weinberg equilibrium, calculated for the Durance in 2001 and 2002. This table was based on the genetic analyses published by Costedoat et al. [42] for the same fish sampling campaign. The hatched box for 2001 corresponds to a mixture of combinations of under-represented genetic markers with one over-represented marker.

Some studies have highlighted the role of shoaling in the dynamics of fish populations [73]–[75]. For the nase, video observations by Ahnelt and Keckeis [76] showed strong competition and territorial fighting between males at mating time. This fighting may favour the formation of groups of individuals of similar size (individuals with the dominant N genome) during mating, potentially accounting for the high likelihood of mating between individuals of the same size, and thus with the same inherited parental mitochondrial type.

Various phenomena (selective mating and nuclear-mitochondrial genome interaction) may account for the hybridisation, hybrid fitness and hybrid combinations observed. The different roles in the observed phenomenon are difficult to quantify. Our findings for this hybrid zone suggest that the frequency of the pure species will decrease, but that two groups—one with traits resembling those of the nase and the other with traits resembling those of the sofie—will be maintained.

### Site and cohort effects

The results obtained revealed a certain variability of ecological traits in the different groups between the different parts of the hybrid zone of the Durance, demonstrating the plasticity of different hybrid combinations. Spatial variation in hybrid zone dynamics has been reported in several species [27], [29], [77], [78]. Various environmental factors may affect the ecological characteristics of fish from the *Chondrostoma* genus. In addition to temperature, habitat characteristics may influence fish growth. Growth seemed to be more rapid at Buech than at the other sites during the first year (except for Avignon nase). Bouchard et al [79] also observed a slower growth for the chub in the Durance at Manosque; they also demonstrated the value of habitat characteristics for the growth of large species. This phenomenon seemed to account for the higher growth rates observed at Buech for nase and HyN hybrids both for growth in the first year and for overall growth.

For the Manosque and Buech sites, the 1998 cohort exhibited growth curves which differed from the group (estimated with all cohorts). Nevertheless, all previous comparisons between groups remained true for this cohort. Environmental factors might impact on fish growth but annual variations did not upset the described group pattern for growth.

Our findings showed a lower level of fitness at the Pertuis site (slower growth and maximal variability despite high temperatures), potentially accounting for the lower frequency of nase and HyN hybrids. This point was also stressed by Costedoat et al [42], who reported that this site seemed to be unfavourable for nase and HyN hybrids. At this site growth of the HyS hybrids was inferior to that of the sofie. However, it is difficult to identify precise causes from the environmental characteristics. These findings showed the plasticity of hybrids and provide evidence of the combined effects of environment and genetics on the phenotype and life history traits of fish.

### Influence of genetics and environment

As reported by Stearns [40], the observed variability between sites showed that genetic and environmental factors control phenotype. Robinson et al [80] reported that 53% of variability was due to the environment and 14% to genetics. In the Durance, in morphometric analysis, the first axis in discriminant analysis accounted for 51.4% of inter-group variability and separated fish as a function of their mitochondrial origin. Genetic information therefore appears to be of prime importance. The capture site of fish began to have an effect from the second axis, accounting for only 16% of inter-group variability, onwards.

Cohort growth variability in the first year can be broken down into variability due to type and variability due to site. For example, at Buech, 42% of variability for nase was due to site, whereas almost 100% of variability for sofie was accounted for by site (Table 2). These findings confirm those of Caumul and Polly [81], who showed that phenotype is the product of phylogenetic history and its recent adaptation to local environments, although the relative importance of these two factors remains unclear.

Growth rate appeared to be more variable than size within a type, indicating a higher plasticity. Estimated values also showed differences between sites for a same type, for example hybrids HyS (Figure 3). Fish growth rate allows size at first reproduction to be adjusted according to environmental variations or species survival strategy [39]. Nevertheless, a range of growth rate from sofie to nase including intermediate hybrids was observed, highlighting a genetic component.

## Supporting Information

Appendix S1 Supplementary: Winbugs Program and growth data of Nase from Buech.
(0.06 MB DOC)

Appendix S2 Supplementary: Growth curves and cohorts effect.
(0.11 MB DOC)

Appendix S3 Supplementary: Morphometry
(1.88 MB DOC)
