# Supplementary material for: Influence of Environment and Mitochondrial Heritage on the Ecological Characteristics of Fish in a Hybrid Zone
Source: PLoS One. 2009 Jun 18;4(6):e5962. doi: 10.1371/journal.pone.0005962 (PMC2693669; doi:10.1371/journal.pone.0005962)
Supplement: Appendix S1 — Supplementary: Winbugs Program and growth data of Nase from Buech. (0.06 MB DOC) [file pone.0005962.s001.doc]

**Supplementary Material**

Influence of environment and mitochondrial heritage on the ecological characteristics of fish in a hybrid zone

**Nicolas Stolzenberg°, Bénédicte Nguyen The, Marie Dominique Salducci, Laurent Cavalli^*^**

Supplementary : Winbugs Program and growth data of Nase from Buech.

I Winbugs Program

# Buech Nase

model

{

for( i in 1 : N ) {

for( j in 1 : T ) {

Y[i , j] ~ dnorm(mu[i , j],tau.c)

mu[i , j] <- Linf*(exp(GRO[i]* (x[j] - FEC))-1) /(exp(GRO[i]*(x[j] - FEC)))

}

GRO[i] ~ dnorm(gro.c,gro.tau)

# cohorte determination

for (k in 1:C) { ecart[k,i] <- (gro.c-GRO[i])*cohorte[k,i]}

}

# estimation of cohort effects and cohort growth curves

for (k in 1:C) { ecartm[k] <- sum(ecart[k,]) /sum(cohorte[k,])}

for (k in 1:C) { for (j in 1:T) { Tm[k,j]<- Linf*(exp((gro.c - ecartm[k])* (x[j] - FEC))-1) /(exp((gro.c - ecartm[k])*(x[j] - FEC)))}}

# estimation of species growth curves

for (j in 1:T) { me[j] <- Linf*(exp(gro.c* (x[j] - FEC))-1) /(exp(gro.c*(x[j] - FEC)))}

# Choice of prior of parameters

FEC ~ dnorm(0.375,576)

Linf~dnorm(500,0.00001)

tau.c ~ dgamma(0.001,0.001)

sigma <- 1 / sqrt(tau.c)

# Choice of prior of random effects mean

gro.c ~ dlnorm(-2,0.6)

# Choice of prior of random effects variances

sigma.gro~ dunif(0,1)

gro.tau <- 1/(sigma.gro*sigma.gro)

}

Data

list(N=122,T=6,C=7,x=c(1.375,2.375,3.375,4.375,5.375,6.375),

cohorte=structure(

.Data=c(0, 0, 0, 0, 0, 0, 0, 0, 0, 0, 0, 0, 0, 0, 0, 0, 0, 0, 0, 0, 0, 0, 0, 0, 0, 0, 0, 0, 0, 0, 0, 0, 0, 0, 0, 0, 0, 0, 0, 0, 0, 0, 0, 0, 1, 0, 1, 0, 0, 0,0, 0, 0, 0, 0, 0, 0, 0, 0, 0, 0, 0, 0, 0, 0, 0, 0, 0, 0, 0, 0, 0, 0, 0, 0, 0, 0, 0, 0, 0, 0, 0, 0, 0, 0, 0, 0, 0, 0, 0, 0, 0, 0, 0, 0, 0, 0, 0, 0, 0, 0, 0, 0, 0,0, 0, 0, 0, 0, 0, 0, 0, 0, 0, 0, 0, 0, 0, 0, 0, 0, 0,

0, 0, 0, 0, 0, 0, 0, 0, 0, 0, 0, 0, 0, 0, 0, 0, 0, 0, 0, 0, 0, 0, 0, 0, 0, 0, 0, 0, 0, 0, 0, 0, 0, 0, 0, 0, 0, 0, 0, 0, 0, 0, 1, 0, 0, 0, 0, 0, 0, 0, 0, 0, 0, 0,0, 0, 0, 0, 0, 0, 0, 1, 0, 0, 0, 0, 0, 0, 0, 0, 0, 0, 0, 0, 0, 0, 0, 0, 0, 0, 0, 0, 0, 0, 0, 0, 0, 0, 0, 0, 0, 0, 0, 0, 0, 0, 0, 0, 0, 0, 0, 0, 0, 0, 0, 0, 0, 0, 0, 0, 0, 0, 0, 0, 0, 0, 0, 0, 0, 0, 0, 0,

0, 0, 0, 0, 1, 0, 0, 0, 0, 0, 0, 0, 0, 0, 0, 0, 0, 1, 1, 1, 0, 1, 1, 0, 0, 0, 0, 0, 0, 0, 0, 0, 1, 1, 1, 1, 1, 1, 1, 1, 1, 1, 0, 1, 0, 1, 0, 0, 0, 0, 0, 0, 0, 1,1, 0, 0, 0, 0, 0, 0, 0, 0, 1, 0, 1, 1, 0, 1, 0, 1, 1, 0, 0, 1, 0, 0, 0, 0, 1, 1, 0, 0, 1, 1, 0, 0, 0, 0, 0, 0, 0, 1, 1, 0, 1, 0, 0, 0, 0, 0, 0, 0, 0, 0, 1, 0, 0,1,1, 1, 0, 0, 0, 0, 0, 0, 0, 0, 0, 0, 0,

0, 0, 0, 1, 0, 0, 0, 0, 1, 1, 0, 0, 0, 0, 0, 1, 1, 0, 0, 0, 1, 0, 0, 1, 0, 0, 0, 0, 0, 1, 0, 1, 0, 0, 0, 0, 0, 0, 0, 0, 0, 0, 0, 0, 0, 0, 0, 0, 0, 0, 1, 0, 1, 0,0, 1, 0, 0, 0, 0, 0, 0, 1, 0, 1, 0, 0, 1, 0, 1, 0, 0, 1, 1, 0, 1, 1, 0, 1, 0, 0, 0, 1, 0, 0, 0, 0, 1, 1, 1, 0, 1, 0, 0, 1, 0, 1, 0, 0, 1, 0, 0, 0, 0, 0, 0, 1, 1,0,0, 0, 0, 0, 0, 0, 0, 0, 1, 1, 1, 1, 1,

1, 1, 1, 0, 0, 1, 0, 1, 0, 0, 1, 1, 1, 1, 1, 0, 0, 0, 0, 0, 0, 0, 0, 0, 1, 1, 1, 0, 1, 0, 1, 0, 0, 0, 0, 0, 0, 0, 0, 0, 0, 0, 0, 0, 0, 0, 0, 1, 1, 0, 0, 1, 0, 0,0, 0, 1, 1, 1, 1, 1, 0, 0, 0, 0, 0, 0, 0, 0, 0, 0, 0, 0, 0, 0, 0, 0, 1, 0, 0, 0, 1, 0, 0, 0, 0, 1, 0, 0, 0, 1, 0, 0, 0, 0, 0, 0, 1, 1, 0, 1, 1, 0, 0, 1, 0, 0, 0,0,0, 0, 0, 0, 1, 0, 1, 0, 0, 0, 0, 0, 0,

0, 0, 0, 0, 0, 0, 1, 0, 0, 0, 0, 0, 0, 0, 0, 0, 0, 0, 0, 0, 0, 0, 0, 0, 0, 0, 0, 1, 0, 0, 0, 0, 0, 0, 0, 0, 0, 0, 0, 0, 0, 0, 0, 0, 0, 0, 0, 0, 0, 1, 0, 0, 0, 0,0, 0, 0, 0, 0, 0, 0, 0, 0, 0, 0, 0, 0, 0, 0, 0, 0, 0, 0, 0, 0, 0, 0, 0, 0, 0, 0, 0, 0, 0, 0, 1, 0, 0, 0, 0, 0, 0, 0, 0, 0, 0, 0, 0, 0, 0, 0, 0, 1, 0, 0, 0, 0, 0,0,0, 0, 1, 1, 0, 1, 0, 1, 0, 0, 0, 0, 0,

0, 0, 0, 0, 0, 0, 0, 0, 0, 0, 0, 0, 0, 0, 0, 0, 0, 0, 0, 0, 0, 0, 0, 0, 0, 0, 0, 0, 0, 0, 0, 0, 0, 0, 0, 0, 0, 0, 0, 0, 0, 0, 0, 0, 0, 0, 0, 0, 0, 0, 0, 0, 0, 0,0, 0, 0, 0, 0, 0, 0, 0, 0, 0, 0, 0, 0, 0, 0, 0, 0, 0, 0, 0, 0, 0, 0, 0, 0, 0, 0, 0, 0, 0, 0, 0, 0, 0, 0, 0, 0, 0, 0, 0, 0, 0, 0, 0, 0, 0, 0, 0, 0, 1, 0, 0, 0, 0,0,0, 0, 0, 0, 0, 0, 0, 0, 0, 0, 0, 0, 0),

.Dim=c(7,122)),

Y=structure(

.Data=c(59.0347585057335, 140, NA, NA, NA, NA, 109.056167557055,

145, NA, NA, NA, NA, 105.774893383509, 155, NA, NA, NA, NA, 67.5601795272406,

140.004696824160, 220, NA, NA, NA, 83.8846948248336, 177.432143645315,

257.369275824597, 315, NA, NA, 68.0944190706397, 105, NA, NA,

NA, NA, 115, NA, NA, NA, NA, NA, 81.233062294672, 135, NA, NA,

NA, NA, 44.9823098594952, 91.5263435565886, 205, NA, NA, NA,

53.9407415911880, 130.596850378876, 220, NA, NA, NA, 54.6383323557624,

105, NA, NA, NA, NA, 65.3049753837965, 150, NA, NA, NA, NA, 93.9305243716065,

130, NA, NA, NA, NA, 69.8623060955951, 140, NA, NA, NA, NA, 70.8176031319167,

120, NA, NA, NA, NA, 67.6236355950836, 150.273118695182, 235,

NA, NA, NA, 48.9655838573018, 128.242868266456, 195, NA, NA,

NA, 72.9569456491393, 162.124790492159, 232.239380061894, 280,

NA, NA, 59.194915355407, 129.771149989082, 218.609046962254,

285, NA, NA, 61.3244417056291, 134.439643587581, 221.133854648853,

290, NA, NA, 53.1870805688709, 118.192232657146, 155, NA, NA,

NA, 49.6713380997570, 125.181596137889, 202.587025309488, 250,

NA, NA, 61.1962361861077, 128.654566718638, 183.138670879777,

226, NA, NA, 54.3313461306467, 120.735017484823, 153, NA, NA,

NA, 45.3475286985596, 93, NA, NA, NA, NA, 48.9392228872499, 133,

NA, NA, NA, NA, 54.8606864194787, 135, NA, NA, NA, NA, 108, NA,

NA, NA, NA, NA, 58.1914048127944, 138, NA, NA, NA, NA, 85.8507231733794,

125.803597301932, 203.593637858378, NA, NA, NA, 69.1562591987088,

110, NA, NA, NA, NA, 60.0354793909462, 98.9883218377699, 137,

NA, NA, NA, 68.3265802151395, 142.525849415019, 236.086413174266,

290, NA, NA, 48.0090679690905, 97.684944648147, 208.469257771484,

270, NA, NA, 79.788714184105, 164.423657020096, 235.532444223594,

300, NA, NA, 68.1662541572531, 158.883838398659, 246.345133382460,

300, NA, NA, 66.5429729342819, 137.896743564543, 206.949892991097,

265, NA, NA, 85.5603278039417, 153.332186911951, 251.740538301871,

300, NA, NA, 47.1308954120331, 122.274125742553, 234.977581661386,

285, NA, NA, 67.8528297034987, 145.702219615366, 240.731463499648,

280, NA, NA, 58.1612955469782, 143.122068120063, 229.941447200886,

285, NA, NA, 94.3518325400665, 175.617159101176, 260.1318895786,

310, NA, NA, 47.1355824145324, 111.781291311518, 204.676330873797,

255.083111937097, 290, NA, 79.7798292458775, 153.315112479521,

229.366302389795, 290, NA, NA, 48.6011882261628, 120.680197295455,

195.302208456570, 278.478847297546, 339.841942104754, 350, 69.0586836684061,

143.110041117821, 234.957835748413, 280, NA, NA, 48.454841279712,

124.169776182411, 220.825531044716, 303.222151340446, 343.884630396262,

390, 74.4122050178478, 120.424628910150, NA, NA, NA, NA, 61.3605460680803,

129, NA, NA, NA, NA, 74, NA, NA, NA, NA, NA, 60.4073359653708,

126.996039619447, 202, NA, NA, NA, 64.2393066689832, 134, NA,

NA, NA, NA, 61.781763327108, 135.442215385815, 212, NA, NA, NA,

70.2761450500522, 161.418355884158, 244.217778908695, 290, NA,

NA, 75.687132835494, 156.846150502763, 235.388401666059, 285,

NA, NA, 57.0032916040114, 109.544807836544, 166.12136075378,

205, NA, NA, 94.2162632777623, 169.660318687823, 215, NA, NA,

NA, 57.0032916040114, 160.524342511996, 205, NA, NA, NA, 77.5914832830784,

127.389572683388, 192, NA, NA, NA, 71.7566486253033, 159.457492652504,

238.904656472353, NA, NA, NA, 83.7329409420981, 155.852205681256,

245.660275141866, NA, NA, NA, 87.5773564190755, 173.251875852244,

280.381327935243, 328.186811990235, 366.123193195413, 385, 79.702091670622,

153.165722023226, 251.467236514817, 305, NA, NA, 77.695240896479,

119.804242483769, 190.035574717458, 241.633655372198, 270, NA,

71.9724657926396, 154.548425806299, 239.623192346186, 286.622352353956,

NA, NA, 54.0515955566153, 142.630374721182, 218.373973436922,

287.913822736092, 320, NA, 75.4050241728418, 111.616079107964,

188.590961883484, 243.94572722091, 268.409406429574, NA, 55.8678085953778,

132.489628166994, 216.030088325857, 245, NA, NA, 62.4363577827999,

155.666313107471, 225.637935897675, 250.284167315104, 265, NA,

69.9731035726207, 157.396487872948, 247.329040507398, 286, NA,

NA, 79.0257093570098, 107.388445992549, 157.364461418026, 211.769444111063,

260, NA, 36.6676648419646, 114.037124893297, 182.811702056806,

224.307779515079, 250, NA, 71.1180904604111, 168.655431418265,

250.247905572702, 275, NA, NA, 62.3209779525082, 140.183907097274,

210.382376138389, 240, NA, NA, 69.05148753122, 117.007327801128,

174.023405761561, 214.751420402799, 250, NA, 69.0485378465068,

132.692491906163, 204.608715960901, 265, NA, NA, 109.811999990950,

184.756997986590, 237.101602461241, 263, NA, NA, 88.7758100706902,

149.079550032196, 229, NA, NA, NA, 50.5477129059837, 122.381930701071,

196.432758375652, 240, NA, NA, 67.7200662212315, 114.751242447590,

170.667960728419, 230.403467759104, 255, NA, 64.9448512876344,

124.806323531257, 192.448428955499, 244.549924452889, 268, NA,

60.3158777071577, 160.134251044348, 241.78395031195, NA, NA,

NA, 54.1900463775245, 121.894307126773, 187.240948379590, 217.227767858392,

NA, NA, 60.4407453527644, 150.690852648106, 223.209943960992,

247.038537499634, 266, NA, 77.5059841770673, 105.323283569615,

191.788432600338, 239.297276629658, 255, NA, 146.859302707190,

210, NA, NA, NA, NA, 41.2520695296834, 126.566774586124, 188,

NA, NA, NA, 84.7740167200568, 118.643235972513, 168.390206567352,

228, NA, NA, 99.0940589203384, 155.325766216878, 205.352232236497,

235, NA, NA, 42.6430940520877, 92.9378053833407, 168.062142594308,

210, NA, NA, 38.5226313456884, 127.854615819310, 185, NA, NA,

NA, 48.6010524318914, 108.001169362438, 184.180342450558, 240,

NA, NA, 71.8637865032729, 132.673175815964, 207.665779778217,

254.961724855921, 281, NA, 62.0975301962787, 113.703236809803,

180.358053324476, 234.727659133985, 266.969727394818, NA, 83.8226937157337,

161.084397357596, 245.307168163064, 291.293611321742, NA, NA,

35.1146146752431, 72.767879187212, 114.345688282824, 149.955553638204,

195, NA, 38.4353204246639, 113.086619720314, 165.714428972249,

208, NA, NA, 58.7592179309087, 165.469125720004, 217, NA, NA,

NA, 70.5354862477505, 155.120059351187, 213, NA, NA, NA, 35.1664222834198,

93.8372579018302, 160.265593088019, 225, NA, NA, 41.5762457138942,

117.080949530122, 169, NA, NA, NA, 38.9879717047659, 139.132884534669,

192, NA, NA, NA, 64.1572710519771, 152.147957823940, NA, NA,

NA, NA, 42.9511680668502, NA, NA, NA, NA, NA, 44.1371083997148,

84.8195064189454, 158.195261361629, NA, NA, NA, 63.0161504423361,

143.801843479608, 232.721011740931, 276.347994427146, 298, NA,

37.7854598595152, 101.624119999156, 167.560935616908, 241, NA,

NA, 46.1795835932629, 124.931554257343, 195.548259655317, 245,

NA, NA, 70.5817985219024, 140.550041254765, 236.973478489169,

288.914691172798, 317, NA, 49.8380206383455, 134.828876637303,

211.039975724598, 243.148925795349, 275, NA, 89.9004228802178,

142.059664124982, 182.922064609818, 213.197436930450, 273, NA,

40.1937476675367, 92.750167549201, NA, NA, NA, NA, 66.3691907908154,

116.586900110024, NA, NA, NA, NA, 45.7566670434493, 98.2545308657642,

133.906916127916, NA, NA, NA, 54.9005944259058, 111.019070835890,

NA, NA, NA, NA, 53.2356280216615, 102.304384143323, 185, NA,

NA, NA, 45.8182988442496, 106.380369824379, NA, NA, NA, NA, 54.3036798021173,

120.673537420429, 195.291430480318, 263, NA, NA, 63.5001799979857,

139.209446172468, 212.342361314301, 273, NA, NA, 59.8156639758291,

104.084186115855, 189.532098099157, 252, NA, NA, 38.2923264863905,

102.178347493104, 179.988230641702, 245, NA, NA, 83.658569131635,

117.082140762348, 182.328322778539, 225, NA, NA), .Dim = c(122,

6)))

Inits

# chain 1 : Nase

list(FEC=0.375,Linf=500,tau.c=1,gro.c=0.26,sigma.gro=0.1)

# chain 2 : Sofie

list(FEC=0.458,Linf=200,tau.c=1,gro.c=0.15,sigma.gro=0.1)

II Buech Nase csv data file

Age;Cohort;Fish Size;Scale Size;Annuli1;Annuli2;Annuli3;Annuli4;Annuli5;Annuli6;Fish Size1;Fish Size2;Fish Size3;Fish Size4;Fish Size5;Fish Size6

2;1999;140;2.5;1.00;2.50;;;;;59.03476;140.00000;;;;

2;1999;145;2.3;1.70;2.30;;;;;109.05617;145.00000;;;;

2;1999;155;3.0;2.00;3.00;;;;;105.77489;155.00000;;;;

3;1998;220;4.2;1.20;2.60;4.20;;;;67.56018;140.00470;220.0000;;;

4;1997;315;5.7;1.40;3.10;4.60;5.70;;;83.88469;177.43214;257.3693;315.0000;;

2;1999;105;1.9;1.20;1.90;;;;;68.09442;105.00000;;;;

1;2000;115;2.1;2.10;;;;;;115.00000;;;;;

2;1999;135;2.4;1.40;2.40;;;;;81.23306;135.00000;;;;

3;1998;205;4.0;0.80;1.70;4.00;;;;44.98231;91.52634;205.0000;;;

3;1998;220;4.0;0.90;2.30;4.00;;;;53.94074;130.59685;220.0000;;;

2;1999;105;2.0;1.00;2.00;;;;;54.63833;105.00000;;;;

2;1999;150;2.9;1.20;2.90;;;;;65.30498;150.00000;;;;

2;1999;130;2.4;1.70;2.40;;;;;93.93052;130.00000;;;;

2;1999;140;2.3;1.10;2.30;;;;;69.86231;140.00000;;;;

2;1999;120;2.1;1.20;2.10;;;;;70.81760;120.00000;;;;

3;1998;235;4.5;1.20;2.80;4.50;;;;67.62364;150.27312;235.0000;

3;1998;195;3.9;0.90;2.50;3.90;;;;48.96558;128.24287;195.0000;

4;1997;280;5.0;1.20;2.80;4.10;5.00;;;72.95695;162.12479;232.2394;280.0000

4;1997;285;5.3;1.00;2.30;4.00;5.30;;;59.19492;129.77115;218.6090;285.0000

4;1997;290;5.2;1.00;2.30;3.90;5.20;;;61.32444;134.43964;221.1339;290.0000

3;1998;155;2.8;0.90;2.10;2.80;;;;53.18708;118.19223;155.0000;

4;1997;250;5.0;0.90;2.40;4.00;5.00;;;49.67134;125.18160;202.5870;250.0000

4;1997;226;4.0;1.00;2.20;3.20;4.00;;;61.19624;128.65457;183.1387;226.0000

3;1998;153;2.7;0.90;2.10;2.70;;;;54.33135;120.73502;153.0000;

2;1999;93;1.5;0.70;1.50;;;;;45.34753;93.00000;;

2;1999;133;2.6;0.90;2.60;;;;;48.93922;133.00000;;

2;1999;135;2.6;1.00;2.60;;;;;54.86069;135.00000;;

1;2000;108;1.8;1.80;;;;;;108.00000;;;

2;1999;138;2.5;1.00;2.50;;;;;58.19140;138.00000;;

3;1998;220;3.8;1.40;2.10;3.50;;;;85.85072;125.80360;203.5936;

2;1999;110;1.8;1.10;1.80;;;;;69.15626;110.00000;;

3;1998;137;2.4;1.00;1.70;2.40;;;;60.03548;98.98832;137.0000;;;

4;1997;290;5.1;1.10;2.40;4.10;5.10;;;68.32658;142.52585;236.0864;290.0000;;

4;1997;270;5.0;0.80;1.70;3.80;5.00;;;48.00907;97.68494;208.4693;270.0000;;

4;1997;300;5.3;1.30;2.80;4.10;5.30;;;79.78871;164.42366;235.5324;300.0000;;

4;1997;300;5.3;1.10;2.70;4.30;5.30;;;68.16625;158.88384;246.3451;300.0000;;

4;1997;265;5.2;1.20;2.60;4.00;5.20;;;66.54297;137.89674;206.9499;265.0000;;

4;1997;300;5.3;1.40;2.60;4.40;5.30;;;85.56033;153.33219;251.7405;300.0000;;

4;1997;285;5.4;0.80;2.20;4.40;5.40;;;47.13090;122.27413;234.9776;285.0000;;

4;1997;280;5.4;1.20;2.70;4.60;5.40;;;67.85283;145.70222;240.7315;280.0000;;

4;1997;285;5.4;1.00;2.60;4.30;5.40;;;58.16130;143.12207;229.9414;285.0000;;

4;1997;310;5.3;1.50;2.90;4.40;5.30;;;94.35183;175.61716;260.1319;310.0000;;

5;1996;290;5.5;0.80;2.00;3.80;4.80;5.50;;47.13558;111.78129;204.6763;255.0831;290.0000;

4;1997;290;5.9;1.50;3.00;4.60;5.90;;;79.77983;153.31511;229.3663;290.0000;;

6;1995;350;6.5;0.80;2.10;3.50;5.10;6.30;6.5;48.60119;120.68020;195.3022;278.4788;339.8419;350

4;1997;280;5.3;1.20;2.60;4.40;5.30;;;69.05868;143.11004;234.9578;280.0000;;

6;1995;390;6.4;0.70;1.90;3.50;4.90;5.60;6.4;48.45484;124.16978;220.8255;303.2222;343.8846;390

2;1999;143;2.4;1.20;2.00;;;;;74.41221;120.42463;;;;

2;1999;129;2.2;1.00;2.20;;;;;61.36055;129.00000;;;;

1;2000;74;1.2;1.20;;;;;;74.00000;;;;;

3;1998;202;3.6;1.00;2.20;3.60;;;;60.40734;126.99604;202.0000;;;

2;1999;134;2.4;1.10;2.40;;;;;64.23931;134.00000;;;;

3;1998;212;3.7;1.00;2.30;3.70;;;;61.78176;135.44222;212.0000;;;

4;1997;290;5.4;1.20;2.90;4.50;5.40;;;70.27615;161.41836;244.2178;290.0000;;

4;1997;285;4.9;1.20;2.60;4.00;4.90;;;75.68713;156.84615;235.3884;285.0000;;

4;1998;205;3.5;0.90;1.80;2.80;3.50;;;57.00329;109.54481;166.1214;205.0000;;

3;1999;215;3.6;1.50;2.80;3.60;;;;94.21626;169.66032;215.0000;;;

3;1999;205;3.5;0.90;2.70;3.50;;;;57.00329;160.52434;205.0000;;;

3;1999;192;3.4;1.30;2.20;3.40;;;;77.59148;127.38957;192.0000;;;

3;1999;265;4.8;1.20;2.80;4.30;;;;71.75665;159.45749;238.9047;;;

3;1999;280;5.4;1.50;2.90;4.70;;;;83.73294;155.85221;245.6603;;;

6;1996;385;7.7;1.60;3.30;5.50;6.50;7.30;7.7;87.57736;173.25188;280.3813;328.1868;366.1232;385

4;1998;305;5.4;1.30;2.60;4.40;5.40;;;79.70209;153.16572;251.4672;305.0000;;

5;1997;270;4.5;1.20;1.90;3.10;4.00;4.50;;77.69524;119.80424;190.0356;241.6337;270.0000

4;1998;297;5.4;1.20;2.70;4.30;5.20;;;71.97247;154.54843;239.6232;286.6224;

5;1997;320;6.6;1.00;2.80;4.40;5.90;6.60;;54.05160;142.63037;218.3740;287.9138;320.0000

5;1997;270;4.8;1.24;1.88;3.28;4.31;4.77;;75.40502;111.61608;188.5910;243.9457;268.4094

4;1998;245;4.8;1.00;2.50;4.20;4.80;;;55.86781;132.48963;216.0301;245.0000;

5;1997;265;5.1;1.10;2.90;4.30;4.80;5.10;;62.43636;155.66631;225.6379;250.2842;265.0000

4;1998;286;4.9;1.10;2.60;4.20;4.90;;;69.97310;157.39649;247.3290;286.0000;

5;1997;260;4.6;1.30;1.80;2.70;3.70;4.60;;79.02571;107.38845;157.3645;211.7694;260.0000

5;1997;250;4.6;0.60;2.00;3.30;4.10;4.60;;36.66766;114.03712;182.8117;224.3078;250.0000

4;1998;275;4.2;1.00;2.50;3.80;4.20;;;71.11809;168.65543;250.2479;275.0000;

4;1998;240;4.6;1.10;2.60;4.00;4.60;;;62.32098;140.18391;210.3824;240.0000;

5;1997;250;4.7;1.20;2.10;3.20;4.00;4.70;;69.05149;117.00733;174.0234;214.7514;250.0000

4;1998;265;5.0;1.20;2.40;3.80;5.00;;;69.04854;132.69249;204.6087;265.0000;

4;1998;263;4.8;1.90;3.30;4.30;4.80;;;109.81200;184.75700;237.1016;263.0000;

3;1999;229;4.1;1.50;2.60;4.10;;;;88.77581;149.07955;229.0000;;

4;1998;240;4.7;0.90;2.30;3.80;4.70;;;50.54771;122.38193;196.4328;240.0000;

5;1997;255;4.9;1.20;2.10;3.20;4.40;4.90;;67.72007;114.75124;170.6680;230.4035;255.0000

5;1997;268;5.4;1.20;2.40;3.80;4.90;5.40;;64.94485;124.80632;192.4484;244.5499;268.0000

3;1999;256;5.1;1.10;3.10;4.80;;;;60.31588;160.13425;241.7840;;

4;1998;230;5.1;1.10;2.60;4.10;4.80;;;54.19005;121.89431;187.2409;217.2278;

5;1997;266;5.3;1.10;2.90;4.40;4.90;5.30;;60.44075;150.69085;223.2099;247.0385;266.0000

5;1997;255;4.6;1.30;1.80;3.40;4.30;4.60;;77.50598;105.32328;191.7884;239.2973;255.0000

2;2000;210;3.8;2.60;3.80;;;;;146.85930;210.00000;;;

3;1999;188;3.5;0.70;2.30;3.50;;;;41.25207;126.56677;188.0000;;

4;1998;228;4.0;1.40;2.00;2.90;4.00;;;84.77402;118.64324;168.3902;228.0000;

4;1998;235;4.5;1.80;2.90;3.90;4.50;;;99.09406;155.32577;205.3522;235.0000;

4;1998;210;3.8;0.70;1.60;3.00;3.80;;;42.64309;92.93781;168.0621;210.0000;

3;1999;185;3.7;0.70;2.50;3.70;;;;38.52263;127.85462;185.0000;;

4;1998;240;4.9;0.90;2.10;3.70;4.90;;;48.60105;108.00117;184.1803;240.0000;

5;1997;281;5.1;1.20;2.30;3.70;4.60;5.10;;71.86379;132.67318;207.6658;254.9617;281.0000

5;1997;283;5.0;1.00;1.90;3.10;4.10;4.70;;62.09753;113.70324;180.3581;234.7277;266.9697

4;1998;305;6.3;1.60;3.20;5.00;6.00;;;83.82269;161.08440;245.3072;291.2936;

5;1997;195;3.7;0.60;1.30;2.10;2.80;3.70;;35.11461;72.76788;114.3457;149.9556;195.0000

4;1998;208;4.2;0.70;2.20;3.30;4.20;;;38.43532;113.08662;165.7144;208.0000;

3;1999;217;4.0;1.00;3.00;4.00;;;;58.75922;165.46913;217.0000;;

3;1999;213;4.2;1.30;3.00;4.20;;;;70.53549;155.12006;213.0000;;

4;1998;225;4.3;0.60;1.70;3.00;4.30;;;35.16642;93.83726;160.2656;225.0000;

3;1999;169;3.1;0.70;2.10;3.10;;;;41.57625;117.08095;169.0000;;

3;1999;192;3.8;0.70;2.70;3.80;;;;38.98797;139.13288;192.0000;;

2;2000;192;3.2;1.00;2.50;;;;;64.15727;152.14796;;;

1;2001;79;2.1;1.10;;;;;;42.95117;;;;

3;1999;163;3.2;0.80;1.60;3.10;;;;44.13711;84.81951;158.1953;;

5;1997;298;5.2;1.00;2.40;4.00;4.80;5.20;;63.01615;143.80184;232.7210;276.3480;298.0000

4;1998;241;5.0;0.70;2.00;3.40;5.00;;;37.78546;101.62412;167.5609;241.0000;

4;1998;245;4.7;0.80;2.30;3.70;4.70;;;46.17958;124.93155;195.5483;245.0000;

5;1997;317;6.4;1.30;2.70;4.70;5.80;6.40;;70.58180;140.55004;236.9735;288.9147;317.0000

5;1997;275;4.9;0.80;2.30;3.70;4.30;4.90;;49.83802;134.82888;211.0400;243.1489;275.0000

5;1997;273;5.2;1.60;2.60;3.40;4.00;5.20;;89.90042;142.05966;182.9221;213.1974;273.0000

2;2000;103;1.9;0.70;1.70;;;;;40.19375;92.75017;;

2;2000;133;2.3;1.10;2.00;;;;;66.36919;116.58690;;

3;1999;149;2.8;0.80;1.80;2.50;;;;45.75667;98.25453;133.9069;

2;2000;122;2.1;0.90;1.90;;;;;54.90059;111.01907;;

3;1999;185;3.0;0.80;1.60;3.00;;;;53.23563;102.30438;185.0000;

2;2000;120;2.5;0.90;2.20;;;;;45.81830;106.38037;;

4;1998;263;4.8;0.90;2.10;3.50;4.80;;;54.30368;120.67354;195.2914;263.0000

4;1998;273;4.7;1.00;2.30;3.60;4.70;;;63.50018;139.20945;212.3424;273.0000

4;1998;252;4.6;1.00;1.80;3.40;4.60;;;59.81566;104.08419;189.5321;252.0000

4;1998;245;4.3;0.60;1.70;3.10;4.30;;;38.29233;102.17835;179.9882;245.0000

4;1998;225;4.0;1.40;2.00;3.20;4.00;;;83.65857;117.08214;182.3283;225.0000
