# Supplementary material for: Influence of Environment and Mitochondrial Heritage on the Ecological Characteristics of Fish in a Hybrid Zone
Source: PLoS One. 2009 Jun 18;4(6):e5962. doi: 10.1371/journal.pone.0005962 (PMC2693669; doi:10.1371/journal.pone.0005962)
Supplement: Appendix S2 — Supplementary: Growth curves and cohorts effect. (0.11 MB DOC) [file pone.0005962.s002.doc]

**Supplementary Material**

Influence of environment and mitochondrial heritage on the ecological characteristics of fish in a hybrid zone

**Nicolas Stolzenberg°, Bénédicte Nguyen The, Marie Dominique Salducci, Laurent Cavalli^*^**

Supplementary : Growth curves and cohorts effect

**1 Group Growth Curves estimated with a Bayesian approach**

N for Nase

HyN for hybrids with nase mitochondrial origin

HyS for hybrids with sofie mitochondrial origin

S for sofie

|  | **Median size** | **and 95%** | **intervals at age :** | |  |  |
| --- | --- | --- | --- | --- | --- | --- |
| **Manosque** | **1** | **2** | **3** | **4** | **5** | **6** |
| **N** |  |  |  |  |  |  |
| 2.5% | 58.28 | 116.6 | 169.2 | 216.9 | 259.5 | 297.2 |
| median | 62.72 | 123.9 | 179.7 | 230.4 | 276.7 | 318.7 |
| 97.5% | 67.38 | 131.0 | 189.2 | 242.8 | 293.0 | 340.5 |
| **HyN** |  |  |  |  |  |  |
| 2.5% | 57.84 | 113.6 | 163.4 | 208.0 | 247.4 | 281.8 |
| median | 61.43 | 119.4 | 171.2 | 217.5 | 258.8 | 295.7 |
| 97.5% | 65.09 | 125.2 | 179.0 | 227.0 | 270.7 | 310.8 |
| **HyS** |  |  |  |  |  |  |
| 2.5% | 50.51 | 100.3 | 143.2 | 180.3 | 211.8 | 238.3 |
| median | 53.85 | 105.9 | 150.7 | 189.4 | 222.6 | 251.3 |
| 97.5% | 57.23 | 111.5 | 158.1 | 198.4 | 233.8 | 265.4 |
| **S** |  |  |  |  |  |  |
| 2.5% | 52.92 | 103.5 | 141.1 | 168.5 | 188.0 | 201.9 |
| median | 54.27 | 105.4 | 143.3 | 171.5 | 192.3 | 207.8 |
| 97.5% | 55.66 | 107.3 | 145.6 | 174.5 | 196.9 | 214.2 |
|  |  |  |  |  |  |  |
| **Buech** | **1** | **2** | **3** | **4** | **5** | **6** |
| **N** |  |  |  |  |  |  |
| 2.5% | 64.12 | 133.5 | 194.3 | 247.9 | 294.6 | 334.9 |
| median | 66.96 | 137.8 | 200.0 | 254.8 | 302.8 | 345.1 |
| 97.5% | 69.85 | 142.1 | 205.7 | 261.6 | 311.3 | 356.2 |
| **HyN** |  |  |  |  |  |  |
| 2.5% | 68.53 | 133.0 | 186.7 | 231.6 | 268.8 | 299.1 |
| median | 72.08 | 138.4 | 193.6 | 239.5 | 277.7 | 309.4 |
| 97.5% | 75.67 | 143.8 | 200.4 | 247.3 | 286.7 | 320.4 |
| **HyS** |  |  |  |  |  |  |
| 2.5% | 54.56 | 98.16 | 134.1 | 163.3 | 186.7 | 205.0 |
| median | 59.4 | 105.4 | 142.7 | 173.0 | 197.6 | 217.5 |
| 97.5% | 64.36 | 112.5 | 151.2 | 182.7 | 209.1 | 231.6 |
| **S** |  |  |  |  |  |  |
| 2.5% | 57.51 | 102.6 | 136.9 | 163.0 | 182.7 | 197.4 |
| median | 59.55 | 105.3 | 140.0 | 166.2 | 186.1 | 201.1 |
| 97.5% | 61.61 | 108.1 | 143.1 | 169.4 | 189.5 | 205.0 |
|  |  |  |  |  |  |  |
| **Pertuis** | **1** | **2** | **3** | **4** | **5** | **6** |
| **HyN** |  |  |  |  |  |  |
| 2.5% | 47.34 | 99.92 | 149.1 | 195.3 | 238.6 | 279.1 |
| median | 51.66 | 107.4 | 160.0 | 209.6 | 256.3 | 300.3 |
| 97.5% | 56.15 | 115.0 | 171.1 | 224.1 | 274.4 | 322.2 |
| **HyS** |  |  |  |  |  |  |
| 2.5% | 55.74 | 105.5 | 145.4 | 173.5 | 193.2 | 206.9 |
| median | 63.61 | 118.2 | 164.4 | 204.0 | 238.1 | 267.5 |
| 97.5% | 72.42 | 130.0 | 181.6 | 229.4 | 273.7 | 314.4 |
| **S** |  |  |  |  |  |  |
| 2.5% | 59.64 | 114.2 | 152.6 | 179.1 | 196.7 | 208.5 |
| median | 61.5 | 116.9 | 155.7 | 182.8 | 201.7 | 215.0 |
| 97.5% | 63.39 | 119.6 | 158.7 | 186.7 | 207.2 | 222.4 |
|  |  |  |  |  |  |  |
| **Avignon** | **1** | **2** | **3** | **4** | **5** | **6** |
| **N** |  |  |  |  |  |  |
| 2.5% | 85.01 | 180.1 | 252.3 | 308.0 | 349.7 | 380.3 |
| median | 89.86 | 186.7 | 260.4 | 316.4 | 358.9 | 391.3 |
| 97.5% | 94.78 | 193.3 | 268.3 | 324.6 | 368.4 | 403.2 |

**2. Cohort effects estimated with a Bayesian approach**

**2.1** The cohort effect on growth rates was defined as the mean differences between all individual growth rates (*Ti)* and group rate (*m_T_)* for each cohort (same group, same date of birth) :

Cohort effect = *(∑^n^_i_ T_i_/n)* − *m_T._*

The scalimetry analysis indicated that the sampled fishes could have borned between 1995 and 2001. It defined potentially 7×2=14 cohorts. Some cohorts were not sampled and others had a low sample size (table S1). When sample size was too low, the estimated cohort effect might not be precise and could be misleading.

The cohort effect was estimated a posteriori using median values and its 95% intervals (see appendix 1 for winbugs program). Results were given in table S2 to S5, the a posteriori non null cohort effect (with probability 95%) were in bold. Most of the non null effects concerned sofie or cohorts with too small sample size to be considered. Overall, the range of the cohort effects were small, they impact only at the hundredth order, whereas group rates *m_T_* were estimated *a posteriori* at a higher level of precision of 10^-1^ (table S6).

| Manosque | 1995 | 1996 | 1997 | 1998 | 1999 | 2000 | 2001 |
| --- | --- | --- | --- | --- | --- | --- | --- |
| N | 0 | 1 | 2 | 1 | 15 | 3 | 1 |
| S | 0 | 3 | 4 | 60 | 69 | 95 | 7 |
| HyN | 0 | 4 | 5 | 20 | 9 | 12 | 2 |
| HyS | 0 | 0 | 7 | 18 | 28 | 19 | 0 |
|  |  |  |  |  |  |  |  |
| Buech | 1995 | 1996 | 1997 | 1998 | 1999 | 2000 | 2001 |
| N | 2 | 2 | 38 | 36 | 34 | 9 | 1 |
| S | 3 | 15 | 48 | 35 | 28 | 8 | 2 |
| HyN | 0 | 2 | 30 | 36 | 24 | 2 | 0 |
| HyS | 0 | 3 | 12 | 9 | 7 | 0 | 0 |
|  |  |  |  |  |  |  |  |
|  |  |  |  |  |  |  |  |
| Pertuis | 1995 | 1996 | 1997 | 1998 | 1999 | 2000 | 2001 |
| S | 0 | 3 | 18 | 56 | 78 | 82 | 3 |
| HyN | 0 | 1 | 3 | 13 | 10 | 1 | 0 |
| HyS | 0 | 0 | 0 | 1 | 7 | 2 | 0 |
|  |  |  |  |  |  |  |  |
| Avignon | 1995 | 1996 | 1997 | 1998 | 1999 | 2000 | 2001 |
| N | 0 | 7 | 14 | 15 | 11 | 13 | 8 |

Table S1 : Sample size for each cohort (same goup, same date of birth).

| **site** | **type** | **cohorts** | **2.5%** | **median** | **97.5%** |
| --- | --- | --- | --- | --- | --- |
| Buech | N | 1995 | **-0.02318** | **-0.01334** | **-0.00539** |
| Buech | N | 1996 | **-0.02894** | **-0.01766** | **-0.008589** |
| Buech | N | 1997 | -0.008361 | -0.003476 | 9.345E-4 |
| Buech | N | 1998 | -4.579E-4 | 0.004024 | 0.008954 |
| Buech | N | 1999 | -0.002368 | 0.002462 | 0.007736 |
| Buech | N | 2000 | -0.01679 | -0.006276 | 0.003283 |
| Buech | N | 2001 | -0.01692 | 0.01777 | 0.05474 |
| Buech | HyN | 1996 | -0.005694 | 0.007033 | 0.01987 |
| Buech | HyN | 1997 | -0.01574 | -0.006346 | 0.002189 |
| Buech | HyN | 1998 | -0.01442 | -0.005521 | 0.003107 |
| Buech | HyN | 1999 | **0.005121** | **0.01434** | **0.02483** |
| Buech | HyN | 2000 | -0.01733 | 0.01263 | 0.04313 |
| Buech | S | 1995 | -0.01915 | -0.002639 | 0.01312 |
| Buech | S | 1996 | **0.0333** | **0.04413** | **0.05586** |
| Buech | S | 1997 | -0.004327 | 0.005386 | 0.01519 |
| Buech | S | 1998 | -0.01044 | -3.017E-4 | 0.0099 |
| Buech | S | 1999 | **-0.03668** | **-0.02505** | **-0.01398** |
| Buech | S | 2000 | **-0.0485** | **-0.0286** | **-0.009501** |
| Buech | S | 2001 | -0.04454 | 0.004005 | 0.0517 |
| Buech | HyS | 1996 | **0.02234** | **0.04183** | **0.06536** |
| Buech | HyS | 1997 | -0.01465 | 0.003406 | 0.02122 |
| Buech | HyS | 1998 | **-0.04179** | **-0.0212** | **-0.003183** |
| Buech | HyS | 1999 | -0.01852 | 0.001214 | 0.02232 |

Table S2 : median cohort effect estimated a posteriori and its 95% intervals for Buech cohorts.

| **site** | **type** | **cohorts** | **2.5%** | **median** | **97.5%** |
| --- | --- | --- | --- | --- | --- |
| Manosque | N | 1996 | **-0.04156** | **-0.0197** | **-0.007209** |
| Manosque | N | 1997 | **-0.02424** | **-0.01079** | **-0.002874** |
| Manosque | N | 1998 | -0.01869 | -0.00576 | 0.003151 |
| Manosque | N | 1999 | -0.00247 | 0.00254 | 0.009638 |
| Manosque | N | 2000 | -0.005723 | 0.002907 | 0.01393 |
| Manosque | N | 2001 | -0.02612 | -0.002899 | 0.01771 |
| Manosque | HyN | 1996 | -0.01413 | -0.004977 | 0.001933 |
| Manosque | HyN | 1997 | -0.002253 | 0.004635 | 0.012 |
| Manosque | HyN | 1998 | **1.985E-4** | **0.00607** | **0.01314** |
| Manosque | HyN | 1999 | -0.01425 | -0.006487 | 2.792E-4 |
| Manosque | HyN | 2000 | -0.01113 | -0.003459 | 0.003761 |
| Manosque | HyN | 2001 | -0.03719 | -0.01329 | 0.007049 |
| Manosque | S | 1996 | **0.004664** | **0.02028** | **0.0346** |
| Manosque | S | 1997 | **0.02512** | **0.03932** | **0.05453** |
| Manosque | S | 1998 | **0.001625** | **0.008847** | **0.01643** |
| Manosque | S | 1999 | -0.00897 | -0.001689 | 0.005638 |
| Manosque | S | 2000 | -0.01329 | -0.006075 | 8.703E-4 |
| Manosque | S | 2001 | -0.03599 | -0.009262 | 0.0164 |
| Manosque | HyS | 1997 | -0.003904 | 0.006496 | 0.01665 |
| Manosque | HyS | 1998 | -0.005035 | 0.004345 | 0.01439 |
| Manosque | HyS | 1999 | -0.01046 | -0.00118 | 0.008207 |
| Manosque | HyS | 2000 | -0.01711 | -0.005295 | 0.00534 |

Table S3 : median cohort effect estimated a posteriori and its 95% intervals for Manosque cohorts.

| **site** | **type** | **cohorts** | **2.5%** | **median** | **97.5%** |
| --- | --- | --- | --- | --- | --- |
| Pertuis | S | 1996 | **0.08083** | **0.1007** | **0.1225** |
| Pertuis | S | 1997 | **0.05156** | **0.06551** | **0.08119** |
| Pertuis | S | 1998 | **0.04358** | **0.05693** | **0.07211** |
| Pertuis | S | 1999 | **4.777E-4** | **0.01205** | **0.02431** |
| Pertuis | S | 2000 | **-0.08232** | **-0.06656** | **-0.05258** |
| Pertuis | S | 2001 | **-0.1201** | **-0.0635** | **-0.01094** |
| Pertuis | HyS | 1998 | -0.07655 | -0.0115 | 0.00931 |
| Pertuis | HyS | 1999 | -0.01263 | 0.002008 | 0.02648 |
| Pertuis | HyS | 2000 | -0.04202 | -0.004096 | 0.01779 |
| Pertuis | HyN | 1996 | -0.01099 | -0.003604 | 0.001518 |
| Pertuis | HyN | 1997 | -0.001499 | 0.003281 | 0.008923 |
| Pertuis | HyN | 1998 | -0.001028 | 0.003083 | 0.008358 |
| Pertuis | HyN | 1999 | -0.00959 | -0.00398 | 3.818E-4 |
| Pertuis | HyN | 2000 | -0.02646 | -0.007492 | 0.008317 |

Table S4 : median cohort effect estimated a posteriori and its 95% intervals for Pertuis cohorts.

| **site** | **type** | **cohorts** | **2.5%** | **median** | **97.5%** |
| --- | --- | --- | --- | --- | --- |
| Avignon | N | 1996 | -0.01062 | 0.00438 | 0.01861 |
| Avignon | N | 1997 | -0.00336 | 0.00938 | 0.02307 |
| Avignon | N | 1998 | -0.01545 | -0.00234 | 0.01059 |
| Avignon | N | 1999 | -0.02625 | -0.01005 | 0.00556 |
| Avignon | N | 2000 | -0.02177 | -0.00612 | 0.00922 |
| Avignon | N | 2001 | -0.01783 | 0.00686 | 0.03203 |

Table S5 : median cohort effect estimated a posteriori and its 95% intervals for Avignon cohorts.

| **site** | **type** | **mean** | **sd** | **MC error** | **2.5%** | **median** | **97.5%** |
| --- | --- | --- | --- | --- | --- | --- | --- |
| Manosque | N | 0.09316 | 0.02016 | 0.001036 | 0.05658 | 0.09233 | 0.1329 |
| Manosque | HyN | 0.1124 | 0.01632 | 8.434E-4 | 0.08104 | 0.1123 | 0.1453 |
| Manosque | S | 0.2988 | 0.01866 | 8.974E-4 | 0.262 | 0.2986 | 0.3351 |
| Manosque | HyS | 0.1488 | 0.01952 | 9.522E-4 | 0.112 | 0.1483 | 0.188 |
| Buech | N | 0.1289 | 0.01283 | 6.593E-4 | 0.1019 | 0.1292 | 0.1527 |
| Buech | HyN | 0.184 | 0.01497 | 6.974E-4 | 0.155 | 0.1838 | 0.2135 |
| Buech | S | 0.2785 | 0.0127 | 4.81E-4 | 0.2543 | 0.2784 | 0.3038 |
| Buech | HyS | 0.2082 | 0.02569 | 0.0011 | 0.1581 | 0.208 | 0.2601 |
| Pertuis | S | 0.3569 | 0.02163 | 0.001019 | 0.3135 | 0.3565 | 0.4005 |
| Pertuis | HyS | 0.1697 | 0.07097 | 0.003507 | 0.07634 | 0.1542 | 0.3422 |
| Pertuis | HyN | 0.05881 | 0.01159 | 5.916E-4 | 0.03903 | 0.05771 | 0.08472 |
| Avignon | N | 0.274 | 0.02166 | 9.079E-4 | 0.2312 | 0.2742 | 0.3164 |

Table S6 : Estimated group rates m_T_.

**2.2** Cohort growth curves estimated with a Bayesian approach

For Manosque and Buech, the cohort growth curves were included in the 95% intervals of the group growth curves at all age (cf. point 1), for cohorts with enough sample size. For Pertuis and Avignon, there were some cohorts affected by lower growth, but this fact did not change their relative size position at four years old with regards to the other type (N, S, HyN and HyS).

|  |  |  | **Median Size at age :** | |  |  |
| --- | --- | --- | --- | --- | --- | --- |
| **Manosque** | **1** | **2** | **3** | **4** | **5** | **6** |
| **N** |  |  |  |  |  |  |
| 1996 | 75.61 | 147.8 | 212.3 | 269.9 | 321.2 | 367.1 |
| 1997 | 69.9 | 137.3 | 198.0 | 252.7 | 301.9 | 346.3 |
| 1998 | 66.63 | 131.2 | 189.7 | 242.6 | 290.4 | 333.7 |
| 1999 | 60.95 | 120.5 | 174.9 | 224.5 | 269.9 | 311.4 |
| 2000 | 60.68 | 120.1 | 174.3 | 223.9 | 269.2 | 310.5 |
| 2001 | 64.69 | 127.7 | 184.9 | 236.8 | 284.0 | 326.6 |
| **HyN** |  |  |  |  |  |  |
| 1996 | 64.03 | 124.2 | 177.6 | 225.1 | 267.4 | 305.0 |
| 1997 | 58.95 | 114.8 | 165.0 | 210.1 | 250.6 | 286.9 |
| 1998 | 58.21 | 113.5 | 163.2 | 207.9 | 248.0 | 284.1 |
| 1999 | 64.84 | 125.7 | 179.6 | 227.5 | 270.0 | 307.8 |
| 2000 | 63.25 | 122.8 | 175.7 | 222.9 | 264.9 | 302.3 |
| 2001 | 68.43 | 132.2 | 188.4 | 237.9 | 281.4 | 319.7 |
| **HyS** |  |  |  |  |  |  |
| 1997 | 51.59 | 101.8 | 145.3 | 183.0 | 215.8 | 244.2 |
| 1998 | 52.35 | 103.1 | 147.1 | 185.2 | 218.1 | 246.6 |
| 1999 | 54.25 | 106.6 | 151.7 | 190.5 | 223.9 | 252.6 |
| 2000 | 55.67 | 109.2 | 155.1 | 194.4 | 228.0 | 256.8 |
| **S** |  |  |  |  |  |  |
| 1996 | 50.97 | 99.88 | 136.9 | 164.9 | 186.1 | 202.2 |
| 1997 | 47.82 | 94.51 | 130.5 | 158.3 | 179.8 | 196.3 |
| 1998 | 52.84 | 103.0 | 140.6 | 168.7 | 189.7 | 205.5 |
| 1999 | 54.54 | 105.9 | 143.8 | 172.0 | 192.8 | 208.3 |
| 2000 | 55.26 | 107.0 | 145.2 | 173.3 | 194.1 | 209.4 |
| 2001 | 55.77 | 107.9 | 146.2 | 174.3 | 194.9 | 210.0 |
|  |  |  |  |  |  |  |
| **Buech** | **1** | **2** | **3** | **4** | **5** | **6** |
| **N** |  |  |  |  |  |  |
| 1995 | 73.52 | 150.3 | 217.0 | 274.7 | 324.8 | 368.3 |
| 1996 | 75.62 | 154.3 | 222.3 | 281.0 | 331.6 | 375.3 |
| 1997 | 68.69 | 141.1 | 204.5 | 260.1 | 308.7 | 351.3 |
| 1998 | 64.96 | 133.9 | 194.8 | 248.5 | 295.9 | 337.7 |
| 1999 | 65.73 | 135.4 | 196.8 | 250.9 | 298.6 | 340.6 |
| 2000 | 70.08 | 143.8 | 208.2 | 264.4 | 313.4 | 356.3 |
| 2001 | 58.04 | 120.4 | 176.2 | 226.2 | 270.9 | 311.0 |
| **HyN** |  |  |  |  |  |  |
| 1996 | 69.5 | 133.9 | 187.9 | 233.1 | 271.0 | 302.7 |
| 1997 | 74.39 | 142.4 | 198.7 | 245.2 | 283.6 | 315.4 |
| 1998 | 74.07 | 141.9 | 198.0 | 244.4 | 282.8 | 314.5 |
| 1999 | 66.83 | 129.2 | 181.8 | 226.3 | 263.8 | 295.4 |
| 2000 | 67.46 | 130.3 | 183.3 | 227.9 | 265.6 | 297.2 |
| **HyS** |  |  |  |  |  |  |
| 1996 | 48.31 | 87.36 | 120.4 | 148.5 | 172.2 | 192.4 |
| 1997 | 58.55 | 104.0 | 141.0 | 171.2 | 195.8 | 215.8 |
| 1998 | 64.88 | 114.0 | 153.0 | 184.0 | 208.6 | 228.2 |
| 1999 | 59.1 | 104.9 | 142.1 | 172.3 | 196.9 | 216.9 |
| **S** |  |  |  |  |  |  |
| 1995 | 60.04 | 106.1 | 140.8 | 167.1 | 186.9 | 201.8 |
| 1996 | 51.15 | 92.23 | 124.7 | 150.5 | 170.8 | 186.9 |
| 1997 | 58.55 | 103.8 | 138.2 | 164.4 | 184.4 | 199.6 |
| 1998 | 59.61 | 105.4 | 140.1 | 166.3 | 186.2 | 201.2 |
| 1999 | 64.16 | 112.3 | 147.8 | 174.0 | 193.3 | 207.6 |
| 2000 | 64.8 | 113.2 | 148.9 | 175.1 | 194.3 | 208.5 |
| 2001 | 58.81 | 104.2 | 138.7 | 164.9 | 184.8 | 199.9 |
|  |  |  |  |  |  |  |
| **Pertuis** | **1** | **2** | **3** | **4** | **5** | **6** |
| **HyN** |  |  |  |  |  |  |
| 1996 | 54.8 | 113.8 | 169.2 | 221.4 | 270.3 | 316.3 |
| 1997 | 48.72 | 101.5 | 151.4 | 198.7 | 243.4 | 285.7 |
| 1998 | 48.9 | 101.8 | 152.0 | 199.4 | 244.2 | 286.7 |
| 1999 | 55.2 | 114.6 | 170.4 | 222.8 | 272.0 | 318.2 |
| 2000 | 58.31 | 120.9 | 179.4 | 234.1 | 285.2 | 332.9 |
| **HyS** |  |  |  |  |  |  |
| 1998 | 68.65 | 126.7 | 175.3 | 216.2 | 251.2 | 280.9 |
| 1999 | 62.33 | 116.0 | 161.6 | 201.0 | 235.0 | 264.4 |
| 2000 | 65.83 | 121.8 | 169.0 | 209.3 | 243.9 | 273.5 |
| **S** |  |  |  |  |  |  |
| 1996 | 45.84 | 91.03 | 126.0 | 153.1 | 174.1 | 190.3 |
| 1997 | 51.45 | 100.6 | 137.3 | 164.8 | 185.3 | 200.7 |
| 1998 | 52.8 | 102.9 | 140.0 | 167.4 | 187.8 | 202.9 |
| 1999 | 59.68 | 114.0 | 152.5 | 179.8 | 199.1 | 212.8 |
| 2000 | 71.17 | 131.6 | 171.1 | 197.0 | 213.9 | 225.0 |
| 2001 | 70.74 | 130.9 | 170.4 | 196.3 | 213.3 | 224.4 |
|  |  |  |  |  |  |  |
| **Avignon** | **1** | **2** | **3** | **4** | **5** | **6** |
| **N** |  |  |  |  |  |  |
| 1996 | 88.54 | 184.4 | 257.6 | 313.4 | 356.1 | 388.6 |
| 1997 | 87.02 | 181.6 | 254.2 | 309.9 | 352.7 | 385.5 |
| 1998 | 90.56 | 188.0 | 261.9 | 318.0 | 360.5 | 392.7 |
| 1999 | 92.83 | 192.1 | 266.8 | 323.0 | 365.2 | 397.0 |
| 2000 | 91.64 | 190.0 | 264.3 | 320.4 | 362.8 | 394.8 |
| 2001 | 87.78 | 183.0 | 255.9 | 311.7 | 354.3 | 386.9 |
