# Supplementary material for: Influence of Environment and Mitochondrial Heritage on the Ecological Characteristics of Fish in a Hybrid Zone
Source: PLoS One. 2009 Jun 18;4(6):e5962. doi: 10.1371/journal.pone.0005962 (PMC2693669; doi:10.1371/journal.pone.0005962)
Supplement: Appendix S3 — Supplementary: Morphometry (1.88 MB DOC) [file pone.0005962.s003.doc]

# Supplementary Material

Influence of environment and mitochondrial heritage on the ecological characteristics of fish in a hybrid zone

**Nicolas Stolzenberg°, Bénédicte Nguyen The, Marie Dominique Salducci, Laurent Cavalli^*^**

Supplementary : Morphometry

Mosimann (1970) [1] proposed explicit measures of size and shape and defined allometry as a dependance of shape on size. In this approach, it is sensible to use a regression of shape on size.This same regression approach can be used with landmark configurations ([2], [3], [4]). (from Klingenberg 2008 course).

With 13 landmarks (coordinate x,y), there was 26 shape variables, with 22 degrees of freedom after procuste superimposition, wich can be related to centroïd size by multivariate regression. Therefore, for each shape variable, we might calculate a shape variable corrected from allometric effects :

Corrected Shape variable = Shape variable – b * Centroïd sizes,

As allometric effects might vary from one type of chondrostoms to the other, multivariate regression were performed independently for each type : nase, hybrids with mitochondrial nase origin, hybrids with mitochondrial sofie origin, sofie. To visualize the strength of association between size and shape, it is desirable to have an analogue of the univariate scatter plot of shape vs size. In multivariate regression, it is possible to compute a regression score that shows the association with centroïd size ([5]) :

Regression score = **shape vector** ***b**^T^/(**bb**^T^)^-0.5^  for each specimen of a type,

were **b** is the vector of the slope of the multivariate regression of shape variables on centroïd sizes for a type of chondrostoms. All types of chondrostoms showed a quite strong relationship as shown in figure S1. Allometric effect seemed non negligeable.

For comparisons of multiple types, there is a danger of confounding true differences between the types with differences in the size compositions. Sofie had a smaller adult size than nase and those differences in size could have driven us to false conclusion. To alleviate this problem, each fish was adjusted to the median centroïd size :

Summary of the centroïd size for all fish from all types :

Min. 1st Qu. Median Mean 3rd Qu. Max.

1083 1916 1966 1956 2016 2272

Thus, within each type, an adjusted shape variable might be calculated :

Adjusted shape variable = Corrected Shape variable + b * 1966.

Eventually, within types, allometric effects are supposed to have been removed with the corrected shape variable. Between chondrostom types the slope vector (**b**) might be different and the allometric effect should remain but adjusted at the same size. Fishes were compared at a same size to alleviate the type differences in adult size. The plot of adjusted shape vs centroïd size showed no more relation of shape with centroïd size within each chondrostom type as expected (figure S2 and S3). Some adjusted shape variables still exhibited differences between types as hoped (figure S2 and S3).

Other variables as sex or sampled sites might have influenced allometric relationship. To account for site influence we considered 13 groups (site × type) instead of four types. The same protocol was applied. The discriminant analysis of adjusted shape variables calculated with thirteen groups was similar to the one realised with the four types . The first axis still discriminate fish with nase mitochondrial heritage from fish with sofie mitochondrial heritage (figure S4).

Summary of axis one score differences between the two discriminant analysis :

Min. 1st Qu. Median Mean 3rd Qu. Max.

-7.426e-01 -1.950e-02 3.242e-04 -3.705e-17 1.966e-02 3.189e-01

Wilcoxon rank sum test between the two dataset of axis one’s score was not significant (W = 632485, p-value = 0.983).

The same approach had been applied to evaluate the sex influence but restricted to the sample fished in year 2001 due to missing information for the year 2002.


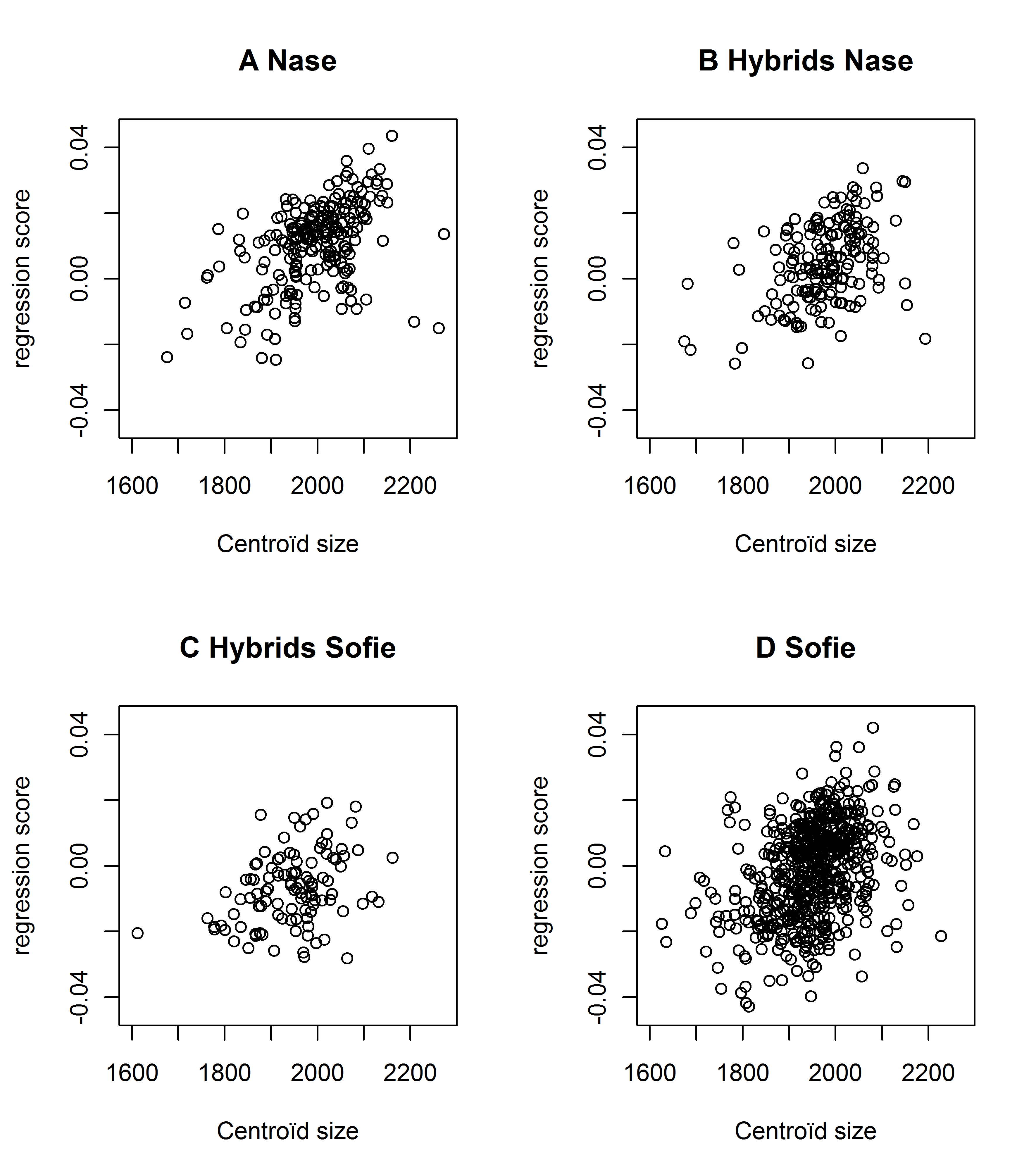


Figure S1 : regression scores vs centroïd size to visualise the allometric relationship of chondrostoms.


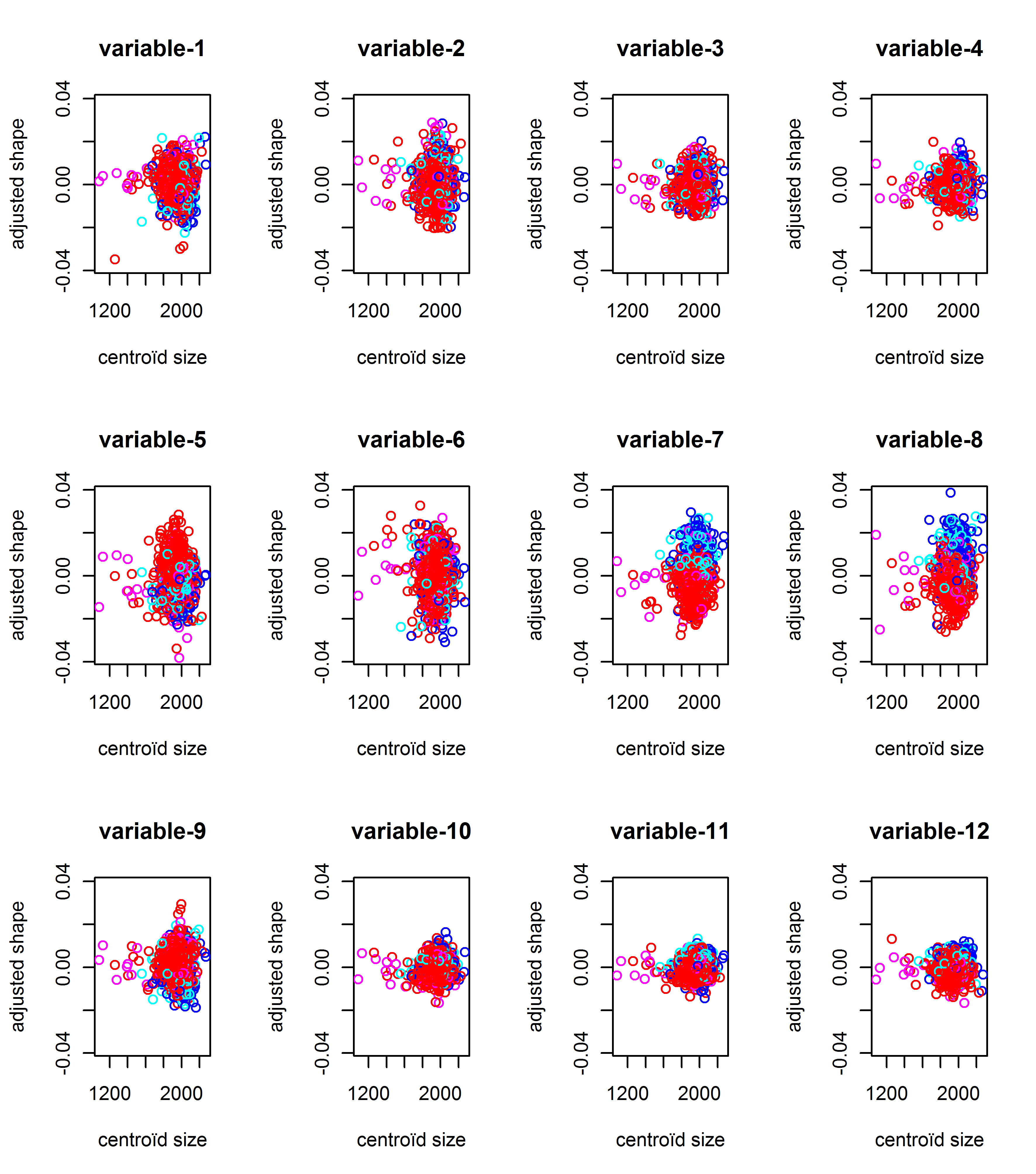


Figure S2 : adjusted shape vs centroïd size for the four chondrostom types. Nase (blue), hybrids Nase (cyan), hybrids sofie (magenta), sofie (red).


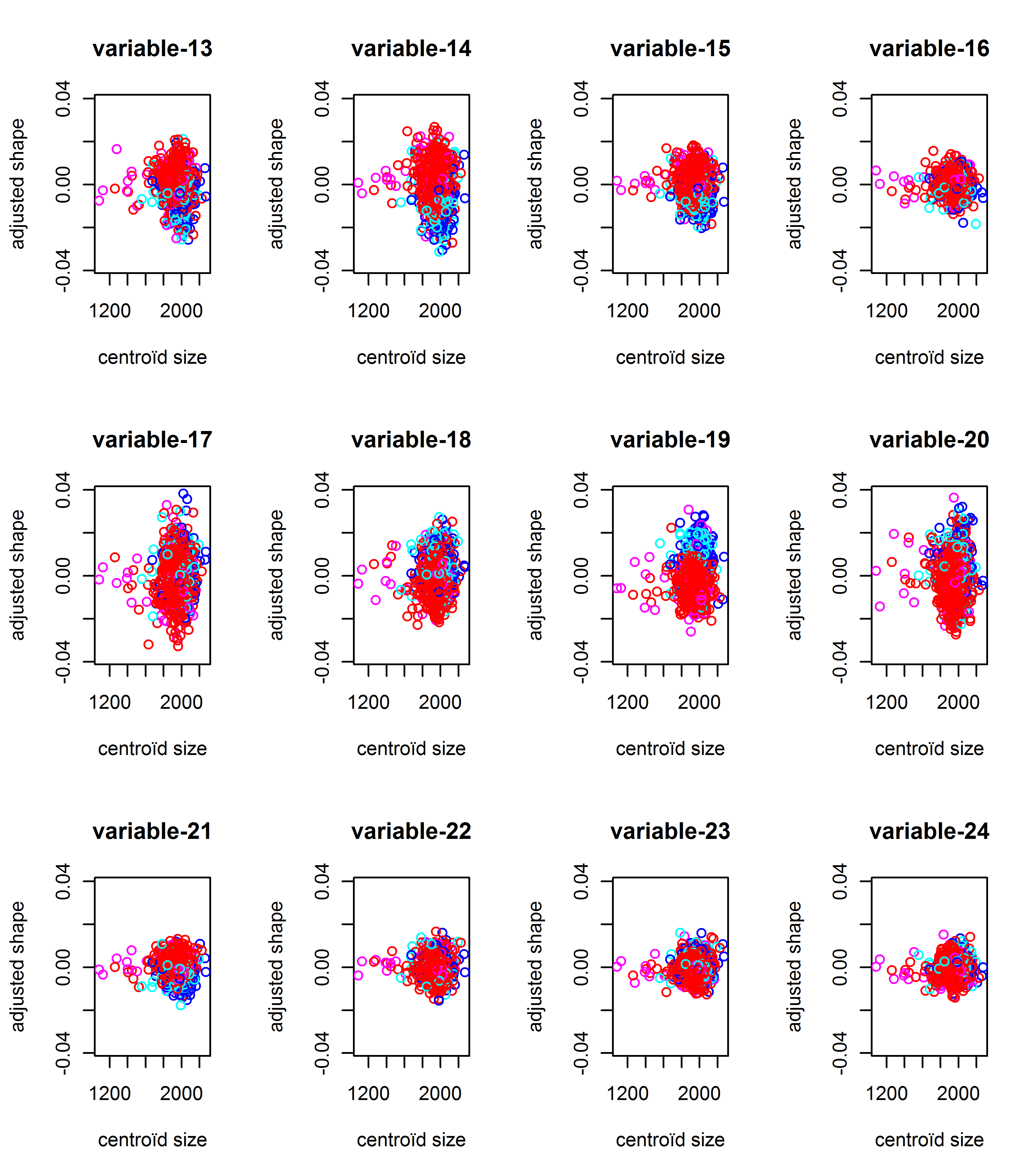


Figure S3: adjusted shape vs centroïd size for the four chondrostom types. Nase (blue), hybrids Nase (cyan), hybrids sofie (magenta), sofie (red).


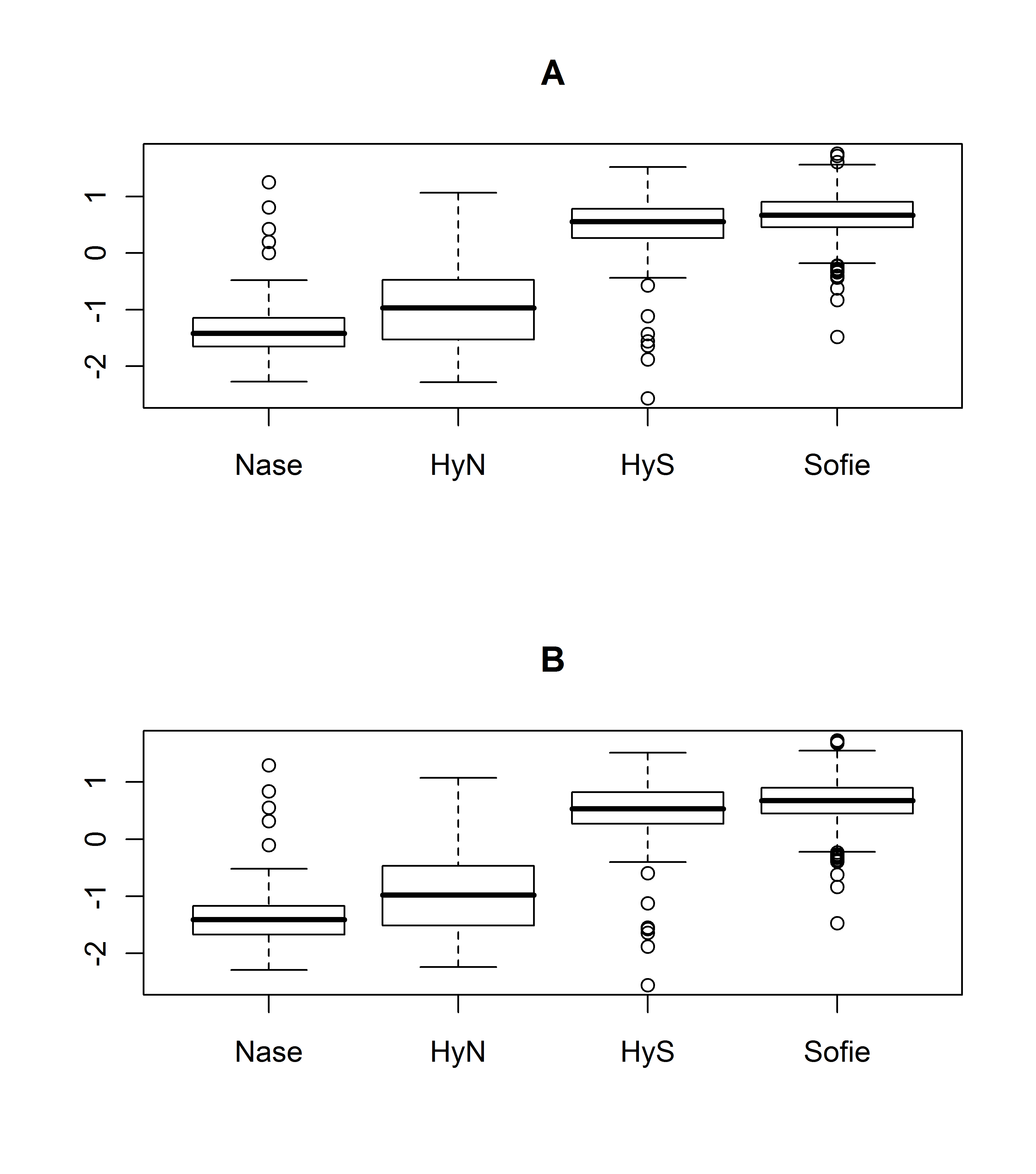


Figure S4 : Boxplots of fish scores on the first discriminant axis. The adjusted shape variables were calculated with four types (A) and with 13 groups (B)

References

1. Mosimann J.E. (1970) Size allometry: size and shape variables with characterizations of the lognormal and the generalized gamma distributions. Journal of the american statistical association 65:930-945

2. Monteiro L.R. (1999) Multivariate regression models and geometric morphometrics: the search for causal factors in the analysis of shape. Systematic biology 48:192-199

3. Penin X.C., Berge C., Baylac M. (2002) Ontogenetic study of ths skull in modern humans and the common chimpanzees: neotenic hypothesis reconsidered with a tridimensionnal Procustes analysis. American journal of physical anthropology 118/50-62

4. Frost,S.R., Marcus,F.L., Bookstein F.L., Reddy D.P., Delson E. (2003) Cranial allometry, phylogeography, and systematics of large-bodied papionins (Primates: Cercopithecinae) inferred from geometric morphometric analysis of landmark data. Anatomical Record 275A/1048-1072

5. Drake,A.G., Klingenberg C.P. (2008) The pace of morphological change: historical transformation of skull shape in st. Bernard dogs. Proceedings of the royal society of London, B biological sciences 275: 71-76
